# A drift-diffusion model of temporal generalization outperforms existing models and captures modality differences and learning effects

**DOI:** 10.3758/s13428-025-02819-8

**Published:** 2025-11-05

**Authors:** Nir Ofir, Ayelet N. Landau

**Affiliations:** 1 Department of Psychology, Hebrew University of Jerusalem, Mt. Scopus, 9190501 Jerusalem, Israel; 2 Department of Cognitive and Brain Sciences, Hebrew University of Jerusalem, Mt. Scopus, 9190501 Jerusalem, Israel; 3 Edmond and Lily Safra Center for Brain Sciences, Hebrew University of Jerusalem, Edmond J. Safra Campus, 9190401 Jerusalem, Israel; 4 Department of Experimental Psychology, University College London, London, WC1H OAP UK

**Keywords:** Behavioral modeling, Psychophysics, Timing and time perception, Drift-diffusion models, Bounded accumulation models, Temporal generalization

## Abstract

Multiple systems in the brain track the passage of time and can adapt their activity to temporal requirements. While the neural implementation of timing varies widely between neural substrates and behavioral tasks, at the algorithmic level, many of these behaviors can be described using drift-diffusion models of decision-making. In this work, wedevelop a drift-diffusion model to fit performance in the temporal generalization task, in which participants are required to categorize an interval as being the same or different compared to a standard, or reference, duration. The model includes a drift-diffusion process which starts with interval onset, representing the internal estimate of elapsed duration, and two boundaries. If the drift-diffusion process at interval offset is between the boundaries, the interval is categorized as equal to the standard. If it is below the lower boundary or above the upper boundary, the interval is categorized as different. This model outperformed previous models in fitting the data of single participants and in parameter recovery analyses. We also used the drift-diffusion model to analyze data from two experiments, one comparing performance between vision and audition and another examining the effect of learning. We found that decision boundaries can be modified independently: While the upper boundary was higher in vision than in audition, the lower boundary decreased with learning in the task. In both experiments, timing noise was positively correlated with upper boundaries across participants, which reflects an accuracy-maximizing strategy in the task.

**Supplementary Information:**

The online version contains supplementary material available at 10.3758/s13428-025-02819-8.

## Introduction

Accurately tracking the passage of time is crucial for all behavior. When we play a ball game, timing is critical from multiple perspectives. From a sensory perspective, we need to constantly track the ball and players and predict their next move. From a motor perspective, we need to time our hands and feet to meet the ball at the right moment. Neurobiological and theoretical studies suggest that implementing the tracking of time takes many different forms, depending on the specific neural network and behavioral goal (Paton & Buonomano, 2018).

Despite this variability in implementation, at the algorithmic level, timing behaviors can often be described as a bounded accumulation process, often called “pacemaker-accumulators” in the timing literature (Balcı & Simen, 2016; Simen et al., 2013). Prominent members of this family include Treisman’s model (Treisman, 1963), scalar expectancy theory (SET; Gibbon, 1977), behavioral theory of timing (BeT; Killeen & Fetterman, 1988), and drift-diffusion models of timing (Simen et al., 2011). We will focus on the application of these models to human timing data and refer the reader to other extensive reviews for further details (Balcı & Simen, 2024; Hass & Durstewitz, 2014).

A central assumption of models of this family is that time is represented in the accumulation of pulses produced by a pacemaker. The simplest demonstration of such models is for tasks where participants are required to produce a certain interval. A variety of designs exist for human participants in the literature for this sort of task, out of which temporal production and reproduction are the most common. In temporal production, participants are asked to produce a single interval of a given duration (e.g., 2.5 s; Kononowicz & Van Rijn, 2011; Macar et al., 1999), while in temporal reproduction, participants are presented with an interval of variable duration on each trial, and are asked to reproduce it (Cicchini et al., 2012).

To the best of our knowledge, pacemaker-accumulator models have not been formally applied to performance in such tasks. A similar task to which they have been applied is the beat-the-clock task (Simen et al., 2011). In this task, participants need to respond as closely as possible to, but not after, a deadline which changes after an unpredictable number of trials. A bounded accumulation model for beat-the-clock assumes that the accumulator resets when the timed interval starts, and that the participant responds when the accumulated value reaches the boundary. Such models could be applied to production and reproduction designs as well.

Bounded accumulation models have been extensively applied to tasks which involve making decisions about elapsed intervals. These tasks include one-interval and two-interval forced-choice designs (1IFC and 2IFC, respectively). In 2IFC, two durations are presented in each trial, and participants are asked to compare them (e.g., decide which interval was longer; Kononowicz & Van Rijn, 2014). In 1IFC, a single interval is presented in each trial, and participants are asked to compare it to a duration, or durations, they learn in the beginning of the experiment. A prototypical example for such a task is temporal bisection, in which participants categorize intervals as being “short” or “long” based on two reference intervals they are familiarized with at the start of the experiment (Allan & Gibbon, 1991; Church & Deluty, 1977; Wearden, 1991).

SET assumes that in bisection, participants compare the number of accumulated pulses to memories of the references and respond according to which reference is more similar to the accumulator value. The drift-diffusion model adapted for temporal bisection is slightly more complex, as it was designed to explain response times in addition to “short”/”long” binary responses (Balcı & Simen, 2014). This model assumes that the accumulator runs until either a decision boundary is reached, at which point the interval is categorized as “long,” or the interval ends. If the interval ends before the accumulator reaches the boundary, a second drift-diffusion model starts in which the accumulator value is compared against an internal bisection point. Neglecting noise, if the accumulator value is larger than the bisection point, the interval is categorized as “long.” If it is below the bisection point, then the interval is categorized as “short.”

A conceptual advantage of the drift-diffusion model is that it naturally incorporates time perception into the larger field of perceptual decision-making (Ratcliff et al., 2016). This unifying perspective is especially useful for studies relating physiology to behavior, as it enables a reliance on the wide range of literature about the neural basis of decision-making (O’Connell & Kelly, 2021). Indeed, temporal decision processes have been found to be reflected by signatures of motor preparation and evidence accumulation (Ofir & Landau, 2022, 2025).

Given the success of the drift-diffusion model in explaining performance and electroencephalography (EEG) in different timing behaviors, we wondered whether it could accommodate other temporal decision tasks as well. An example of such a task is the temporal generalization task, introduced to human research by John Wearden (1992). In this task, participants are required to decide whether an interval is the same as a previously presented standard.

Temporal generalization remains a relatively understudied experimental design. We believe one reason is the lack of easy-to-use established models. This is in contrast to other more common designs, such as temporal bisection or discrimination, which yield sigmoid data that can be analyzed using widely available toolboxes for psychophysical data such as Psignifit (Schütt et al., 2016) and Palamedes (Prins & Kingdom, 2018).

Each experimental design is useful as it emphasizes different parts of the cognitive process. Temporal reproduction emphasizes motor timing components, while temporal generalization emphasizes perceptual decision-making aspects of timing behavior. Describing timing performance in different tasks at a cognitive level is necessary to arrive at a complete picture of how animals compute and use time. The complexity of timing at the neural level underscores the importance of studying time using many designs, as how timing is carried out in one task does not generalize to other tasks (Paton & Buonomano, 2018).

Our work has several methodological goals. First, we summarize three existing models of temporal generalization as well as a new one and provide code for fitting all models using a maximum likelihood approach. Current models have never been thoroughly tested and compared, and previous work with human participants only fit models to data pooled across participants (Birngruber et al., 2014; Droit-Volet et al., 2001). Due to interindividual variability, group data are often not representative of behavior at the single participant level (Ratcliff, 1979), and are suboptimal for testing models which are meant to apply to individuals. To fill this gap, we compare the parameter recoverability of the different models and their fit to data of single participants. We also consider model recoverability—how accurately can we estimate which model of the set considered generated a dataset.

At the empirical level, we conduct two experiments. Beyond enabling the comparison of models on empirical data, these experiments provide a useful test for the models as tools to compare behavior in different conditions. The first compares temporal generalization in the visual and auditory modalities. Auditory timing performance is known to be better than visual timing (Cicchini et al., 2012; Di Luca & Rhodes, 2016; Espinoza-Monroy & De Lafuente, 2021; Wearden et al., 1998). The temporal generalization design accompanied by a cognitive model allows for testing differences between modalities at the levels of perception and decision-making. The second experiment explores the effect of learning in the task. Hypothetically, learning could manifest as an improvement in timing accuracy as well as a difference in decision-making aspects (Masís et al., 2023).

### Temporal generalization

**Fig. 1:**
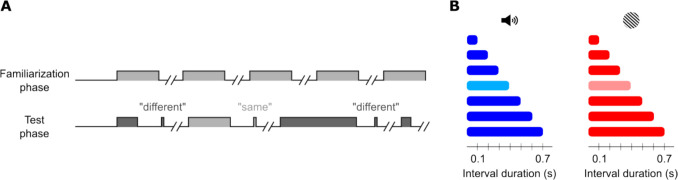
Schematic representation of a temporal generalization task. **A** Progression of the familiarization and test phases. While the familiarization phase is passive, after each interval in the test phase, the participant must respond before the experiment continues. **B** A graphical summary of experiment 1, a temporal generalization experiment designed to compare performance in the auditory (blue) and visual (red) modalities. This design includes seven probe durations (including the standard 0.4 s, in lighter colors)

Before delving into the models, we introduce the structure of a temporal generalization experiment. A typical temporal generalization experiment consists of several blocks of trials with an identical structure (Fig. 1A). Each block starts with several repetitions of the standard duration, which participants are not requested to respond to (familiarization phase). Next, the test phase starts. In each trial of this phase, the participant is presented with a single interval and is asked to report whether the interval had the same duration as the standard or not. The presented duration varies from trial to trial, and usually covers a range spread evenly around the standard (Fig. 1B). Throughout the text, we will use “same” to indicate participants’ judgement of an interval as equal to the standard, while we will retain “standard” for describing the actual duration of an interval.

### Modeling temporal generalization performance

**Fig. 2:**
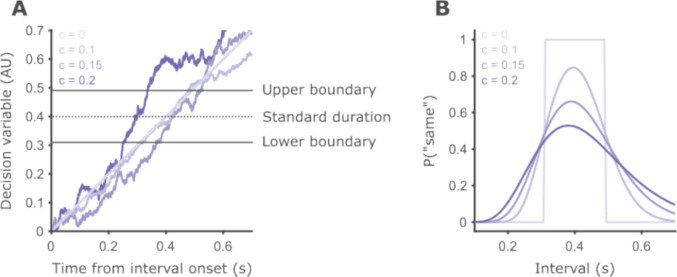
Schematic representation of the drift-diffusion temporal generalization model. **A** The basic components of the model—decision boundaries, set at 0.31 and 0.49, and decision variable—and examples of the evolution of the decision variable over time. Darker hues correspond to traces simulated with larger diffusion coefficients. For the hypothetical case of no noise, the decision variable grows linearly as a function of time with a slope of 1. The traces become more jagged as the diffusion coefficient increases. **B** Psychometric curves for the decision boundaries and three levels of diffusion coefficient plotted in **A**. When there is no noise, the curve is two step functions, and the location of the steps is determined by the boundary parameters. For the given decision boundaries and the intervals we used, this model will produce 100% accurate responses. As internal noise grows, the curves become wider with shallower slopes, and the asymmetry increases.

Temporal generalization performance is typically summarized by plotting a psychometric curve—the probability of “same” responses as a function of interval duration (Fig. 2B). This function is also sometimes referred to as the generalization gradient. From short to long intervals, the probability of a “same” response increases smoothly towards a maximum at or close to the standard duration, and then decreases for longer intervals. The curves are typically asymmetric around their maximum, with the rising part of the curve (i.e., for intervals shorter than the standard) steeper than the falling part. These are the basic properties all models of temporal generalization must display. Beyond that, approaches vary in which aspects they focus on—theoretical or methodological (Wilson & Collins, 2019). For example, SET-based approaches emphasize that models should show “scale invariance”—multiplying the intervals in a task by a factor should yield superimposed psychometric curves (Church & Gibbon, 1982). In contrast, in this work we emphasize the use of models to summarize behavior into cognitively meaningful parameters as a tool to study behavior in different conditions (Wichmann & Jäkel, 2018). The two aspects are not mutually exclusive, and we do not believe they should compete for importance. Efficient summaries of a given behavior require a theoretical understanding of that behavior, and theories of behaviors must also provide adequate fits of empirical data.

All models considered here rely on a common approach in models of perception: a decision variable (DV) sample is drawn on each trial, and this sample is compared against two decision boundaries to produce a binary decision: “same” if the DV is within the boundaries, “different” otherwise. The models differ along several dimensions: whether decision boundaries are constrained to be symmetric around the true standard duration, whether there is trial-to-trial variability in the boundaries, and how noise in the internal representation of the current interval depends on the interval duration. In this work we focus on cognitive models for temporal generalization. Analytical approaches that do not assume a specific cognitive model were reviewed recently elsewhere (Bausenhart et al., 2018; See also Piras & Coull, 2011).

### *Church* & Gibbon, 1982* (CG model)*

Russell Church and John Gibbon originally developed a model to describe the performance of rats in a temporal generalization task (Church & Gibbon, 1982; henceforth CG, following Wearden, 1992). The model assumes that on each trial, a random sample of the standard duration is retrieved from memory as well as a random sample of the decision boundary. Then, the absolute value of the normalized difference of the current duration, which is assumed to be accurately perceived, and the sample of the standard is computed. This normalized difference is compared against the decision boundary. If the absolute normalized difference is smaller than the boundary, the interval is categorized as “same,” and if it is larger than the boundary, the interval is categorized as “different.” The decision rule can be formalized as follows:

$$-b<\frac{t-s}{s}<b$$

where *s* (the standard memory) and *b* (the decision boundary) are both normally distributed random variables, and *t* is equal to the duration presented on the current trial. The psychophysical function—the probability of labeling an interval *t* as “same”—was derived by Church and Gibbon as

$$\begin{array}{c}P\left({\mathrm{same}}|t;B,k,{\sigma }_{B}\right)=\Phi \left({z}_{2}\right)-\Phi \left({z}_{1}\right)\\ {z}_{i}=\frac{\left(1+{\left(-1\right)}^{i}B\right)-\frac{t}{S}}{\sqrt{{k}^{2}{{\sigma }_{B}}^{2}+{k}^{2}{\left(1+{\left(-1\right)}^{i}B\right)}^{2}+{{\sigma }_{B}}^{2}}}\end{array}$$

where $\Phi$ is the standard normal cumulative density function and *S* is the true standard duration. The model has three free parameters: *B* (mean boundary value, relative to the standard), ${\sigma }_{B}$ (boundary standard deviation), and $k$ (Weber fraction, which specifies the variability of standard memory samples).

### *Wearden,*^1992^* (MCG: modified Church & Gibbon model)*

Later work that developed an analogue of the experimental design for humans found that humans displayed greater asymmetries in their psychophysical curves compared to rats (Wearden, 1992; henceforth MCG “modified Church & Gibbon”). Wearden suggested modifying the DV, replacing the standard memory sample in the denominator with the objective duration of the current interval (the variables are the same as in the original model by Church & Gibbon):

$$-b<\frac{t-s}{t}<b$$

The psychophysical function can be found by algebraic operations (Appendix 1) as

$$\begin{array}{l}P\left({\mathrm{same}}|t;B,k,{\sigma }_{B}\right)=\Phi \left({z}_{2}\right)-\Phi \left({z}_{1}\right)\\ {z}_{i}=\frac{t\left(1+{\left(-1\right)}^{i}B\right)-S}{\sqrt{{t}^{2}{{\sigma }_{B}}^{2}+{S}^{2} {k}^{2}}}\end{array}$$

The free parameters are the same as for the CG model. Normalizing the DV by the interval duration instead of the standard means that the variability of the DV decreases for longer durations. This property is unusual for timing models, which typically assume that the uncertainty of estimated duration increases for longer intervals (Hass & Durstewitz, 2016). Over the years, many different variants of this model were developed to fit different scenarios, such as changes in performance over development (Droit-Volet et al., 2001; Wearden, 2004). We focus on the simplest one, as it provides the clearest comparison to the other models.

### *Birngruber,* Schröter, & Ulrich, 2014* (BSU model)*

Both CG and MCG models assume that participants place their decision boundaries symmetrically around the true standard duration. This assumption seems too strict, for two reasons: First, individual participants often display idiosyncratic biases, in timing and other forms of perception, that result in shifted psychometric curves (Gibbon et al., 1984; Lebovich et al., 2019). Second, some experimental manipulations can create systematic shifts in the psychometric curves across participants. The third model we review was developed for data representing such a scenario. Birngruber and colleagues found that when the comparison interval is an oddball in a sequence of stimuli, it is perceived as longer than its objective duration (Birngruber et al., 2014; henceforth BSU). Specifically, the peak of the psychometric function, which is the interval that is most often reported to be equal to the standard, is shorter than the standard. To allow the model to capture such biases, the authors suggested the following psychophysical function:

$$P\left({\mathrm{same}}|t;{b}_{1},{b}_{2},\varepsilon ,k\right)=\Phi \left(\frac{{b}_{2}-\left(t+\varepsilon -s\right)}{kt}\right)-\Phi \left(\frac{{b}_{1}-\left(t+\varepsilon -s\right)}{kt}\right)$$

where *s* and *t* are the objective standard and comparison intervals, respectively, $k$ is the Weber fraction, which describes how quickly noise grows with the comparison interval, ${b}_{1}$ and ${b}_{2}$ are the lower and upper boundaries, respectively, and $\varepsilon$ represents a bias term. We make two technical notes. First, as *s* is the objective standard duration, its only effect is that the boundaries are expressed as relative to the standard rather than in absolute terms. That can be done independently of the fitting procedure if desired. Second, $\varepsilon$ trades off perfectly with the boundaries. For any choice of $\varepsilon$, we can define new boundaries ${b}_{1}{\prime}={b}_{1}-\varepsilon , {b}_{2}{\prime}={b}_{2}-\varepsilon$ which would undo the effect of $\varepsilon$. In the original work, the value of the bias was constrained by additional data from a separate temporal bisection task. However, when only data from a temporal generalization task is available, this function is over-parameterized, and not all parameters can be estimated (see also Appendix 2 in Birngruber et al., 2014). Hence, we remove $\varepsilon$ and *s* from the function, yielding the simplified form

$$P\left({\mathrm{same}}|t;{b}_{l},{b}_{u},k\right)=\Phi \left(\frac{{b}_{u}-t}{kt}\right)-\Phi \left(\frac{{b}_{l}-t}{kt}\right)$$

This model essentially states that a noisy estimate of the comparison interval is compared to two boundaries. If it is within those boundaries, it is reported as “same,” and as “different” otherwise. The model has three free parameters: the upper (${b}_{u}$) and lower (${b}_{l}$) boundaries and the Weber fraction *k*.

### Proposed drift-diffusion model (DDM)

Previous research showed that the drift-diffusion framework captures behavioral as well as different aspects of neural activity in the temporal bisection task (Balcı & Simen, 2014; Ofir & Landau, 2022). We propose a modified drift-diffusion model (henceforth DDM), derived from this framework, for the temporal generalization task. The proposed model includes a single drift-diffusion process with two boundaries. Unlike the typical implementation of the DDM in two-choice scenarios (Ratcliff et al., 2016), here both boundaries are placed above the starting point of the drift diffusion process. At interval onset, the drift-diffusion process starts, and the accumulated value is compared to the boundaries at interval offset. If the accumulated value at the offset has not reached the lower boundary or has surpassed the upper boundary, the interval is categorized as “different.” Otherwise, if the accumulated value at the offset is between the two boundaries, the interval is categorized as “same.” The psychophysical function is as follows (see Appendix 2 for the mathematical derivation):

$$p\left(\mathrm{same}\vert t;b_l,b_u,c\right)=\Phi\left(\frac{b_u-t}{c\sqrt t}\right)-\Phi\left(\frac{b_l-t}{c\sqrt t}\right)$$

The model has three free parameters: the diffusion-to-drift ratio *c*, controlling how rapidly noise grows with time, the ratio of the lower boundary to drift (${b}_{l}$), and the ratio of the upper boundary to drift (${b}_{u}$). For brevity, the parameters will be denoted as diffusion coefficient and lower and upper boundary. We note that the DDM and BSU are very similar. They differ only in how rapidly timing noise grows with interval duration. The faster growth in variability assumed by BSU translates into curves that are generally more asymmetric than those produced by the DDM.

### Summary of models

To summarize, all models assume a decision variable which is compared against decision boundaries. Taking the DDM as an example, we plot simulations of a single trial with different levels of internal noise to visualize how the decision variable dynamically evolves (Fig. 2A). We can also think about the models through the psychometric curves they produce (Fig. 2B). All four models have parameters that control the slopes (rising and falling) and asymmetry of the curve, which reflect the internal noise in the perceptual decision process. While the DDM and BSU models restrict noise to originate only from the timing process, CG and MCG assume that both timing (through the memory of the standard) and decision variability affect the slopes. In addition, DDM and BSU can produce curves that are not centered on the true standard, while CG and MCG cannot.

## Simulation methods

The data and code for all analyses are available at https://osf.io/87zbp/. The code for the simulation analyses is found in “recovery_script.m.”

### Fitting the model to behavior

The three free parameters of each model were estimated by a numerical maximum likelihood procedure, similarly to fits of other psychometric functions (Prins & Kingdom, 2018). First, the probability of a “same” response for each duration was calculated for a given set of parameters. Then, the logarithms of the probabilities were summed for all trials of a single participant in a single condition. The set of parameters that attained the maximum likelihood was found numerically by the Nelder–Mead algorithm, as implemented in the fminsearch function of MATLAB (MathWorks, MA).

When working on the fits of the CG and MCG models, we noticed that fminsearch would sometimes try combinations of the two slope parameters that led to imaginary numbers in the denominator of the psychometric function, which caused MATLAB errors. Therefore, for MCG and CG specifically, we constrained all parameters to be positive using fmincon.

For all models, we initialized the numerical search from eight starting points in the parameter space (all combinations of two values for each of the three parameters). The values are described in Table 1. These values were chosen such that the fitting procedure would start from several types of curves: nearly flat to very narrow as well as shifted horizontally, in the case of DDM and BSU, which allow for that. These values were also chosen as they produce finite likelihoods (Wilson & Collins, 2019). For CG and MCG, as the models operate on durations normalized by the standard, we used specific numbers. Decision boundaries for BSU and DDM were chosen based on the range of intervals in the experiment.

**Table 1:** Initial guesses for the different parameters and models

CG and MCG	BSU	DDM
$B$= 0.1 or 0.5	${b}_{l}$= 25% above the shortest duration or the mean duration	${b}_{l}$= 25% above the shortest duration or the mean duration
$k$= 0.05 or 0.5	${b}_{u}$= 25% below the longest duration or the mean duration	${b}_{u}$= 25% below the longest duration or the mean duration
${\sigma }_{B}$= 0.05 or 0.5	$k$= 0.1 or 0.5	$c$= 0.05 or 0.5

### Parameter recovery

An important step in testing a model is examining its ability to fit the data it simulated. This is called parameter recovery, and it measures the fitting capability of the model under an ideal situation (Wilson & Collins, 2019). In a parameter recovery analysis, a dataset is simulated by a model with given parameters, and then the model is fitted to the simulated data to estimate the model’s parameters. If the model parameters are well defined and the data collection is well suited, we expect that the estimated parameter values will be close to the values used to simulate the data.

To ensure the parameter values tested represented values observed in real behavior, we fitted probability distributions to the parameter values estimated from participants’ behavior in the two experiments we conducted (Fig. S1). This was done for each parameter separately (12 independent distributions in total, three for each of the four models). Each participant in each condition was treated as independent datapoints. To control for the influence of outliers on parameter recovery, we removed datapoints in which any of the parameters was more than three times the interquartile range (IQR) away from the median. In all, between 8 and 35 datapoints were removed for each model, resulting in between 150 and 177 datapoints per parameter for each model. We chose the distributions manually to reasonably fit the parameter values, while only simulating non-negative values. Gamma distributions were fitted to all parameters of the DDM and BSU models, as well as the boundary separation parameter of the CG and MCG models. Because of the large number of noise parameters close to zero in the CG and MCG (see Experiment results), we used exponential distributions for both boundary and timing variability parameters in those models.

For each model, 5,000 simulations were generated from the parameter distributions using the same trial numbers as in a single modality in experiment 1 (see Experimental methods). The simulated parameters were independent, except for a few cases, which would have caused issues later on in fitting the simulated data. For DDM and BSU, upper boundaries must be sufficiently larger than lower boundaries; otherwise the model labels all intervals as “different.” To prevent such cases, we redrew parameters in which the upper boundary was less than 0.1 larger than the lower boundary.

Finally, the results of each simulation were fitted by the model that created it, and the estimated and simulated parameters were compared. A fraction of the simulations resulted in fits with very large parameter estimates, far from the group. Hence, we removed all simulations in which the estimated parameter value was larger than five times the largest simulated value. This resulted in removing less than 3.5% of simulations for each model. As a general measure for fitting accuracy, we calculated the Pearson correlation coefficient between simulated and estimated parameter values. Parameter trade-off was assessed using the correlation between all pairs of estimated parameter values.

### Model recovery

Parameter recovery aids in understanding how reliably we can estimate model parameters from data. Another relevant question is how well we can estimate which of the four models generated the data. This is especially important when comparing the models in terms of their ability to fit data. This analysis is called model recovery. To estimate model recovery in our setting, for each model, we simulated 1,250 datasets using the same parameter distributions as in the parameter recovery analysis, resulting in a total of 5,000 simulated datasets. We then fitted each simulation using each of the four models. Finally, we counted the number of simulations that were best fitted by each model to compute a confusion matrix.

## Simulation results

### Parameters of the DDM are recovered more successfully than the other models

Good computational models need to be identifiable: a given set of data should be captured by a unique set of parameter values. This can be tested by analyzing parameter recovery, or the accuracy of fitted parameters as estimates of the values which generated the data (Wilson & Collins, 2019). We used the parameter values we estimated in empirical data to generate synthetic participants for which we knew the ground truth.

**Fig. 3:**
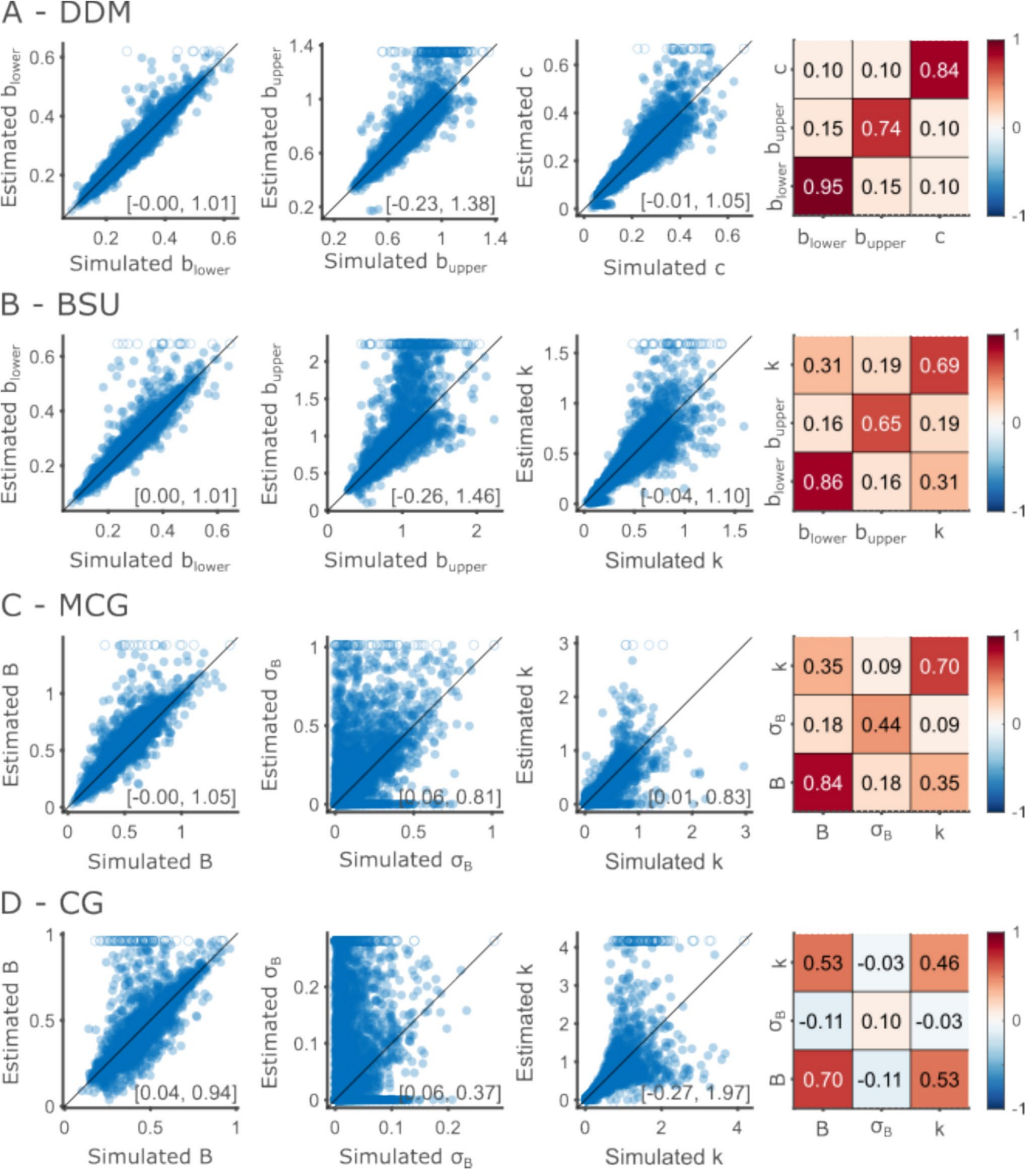
DDM parameters are more accurately recovered than the other models. Each row shows the result of one model. **A** Parameter recovery for the DDM. Scatter plots show the estimated parameter (*y*-axis) against the simulated parameter (*x*-axis) for each of the free parameters (left to right: lower boundary, upper boundary and diffusion). Each point represents a single simulation. To facilitate visualization, values that were estimated as larger than the largest simulated parameter are truncated and replaced with empty circles. The numbers at the bottom right of each scatter plot represent the intercept and slope of the regression of estimated and simulated parameters. The heatmap at the right shows the parameter correlations. Diagonal cells depict the correlation coefficient between simulated and estimated parameters, and off-diagonal cells depict the correlation between the estimated values of two different parameters. **B** Same as **A**, for the BSU model. Scatter plots depict, left to right, the lower boundary, upper boundary, and Weber fraction. **C** Same as **A**, for the MCG model. Parameter scatter plots show, left to right, the mean boundary, boundary standard deviation, and Weber fraction. **D** Same as **A**, for the CG model. Parameter scatter plots show, left to right, the mean boundary, boundary standard deviation, and Weber fraction.

We found that the DDM model displayed the overall highest recovery accuracy for all three parameters (Fig. 3A). Upper boundaries were generally estimated well, up to upper boundaries of about 0.8 s. Above this point, the fitting procedure tended to inflate the estimated upper boundaries. This means that upper boundary estimates larger than 0.8 should be treated cautiously. The experimental design explains this finding: The longest interval we tested is 0.7, meaning that upper boundaries beyond that point are difficult to estimate. Cross-parameter correlations were low overall, estimated at 0.1 for the correlation between noise and lower and upper boundaries, indicating good parameter identifiability. The correlation coefficient between upper and lower boundaries was estimated to be 0.15. The simulated boundaries were constrained to have a separation of at least 100 ms, which resulted in a correlation of 0.11 between lower and upper simulated boundaries. Some of the correlation seen in the estimated values is probably driven by the correlation in the simulated values.

The BSU model’s parameters were recovered somewhat less successfully (Fig. 3B). This is most apparent in the estimation of upper boundaries in that model. The variability of estimated upper boundaries increased quite rapidly with increasing simulated upper boundaries. This translates into lower recoverability, as measured by the correlation between simulated and estimated upper boundaries. We note that the range of upper boundaries estimated in our data is larger for the BSU than the DDM. This results in more upper boundaries simulated to be above 1, values which are difficult to estimate given the intervals we used.

The parameters of the MCG and CG models were least successfully recovered (Fig. 3C, D). Mean boundary separation was recovered relatively well. However, both noise parameters—boundary variability and timing variability—were not estimated accurately. Boundary variability was especially difficult to estimate, most clearly so for the CG model, where estimated values are spread far from the diagonal. Timing variability was recovered more accurately.

In summary, the DDM parameters were recovered well for the range of parameters we estimated in empirical data. The BSU parameters were recovered almost as well. The CG and MCG noise parameters were generally not recovered accurately, reflecting a trade-off between the two. Given the number of trials we have in a single modality, it is not possible to say whether variability in performance stems from noise in the boundaries or in timing.

### All four models are recoverable, with BSU leading

**Fig. 4:**
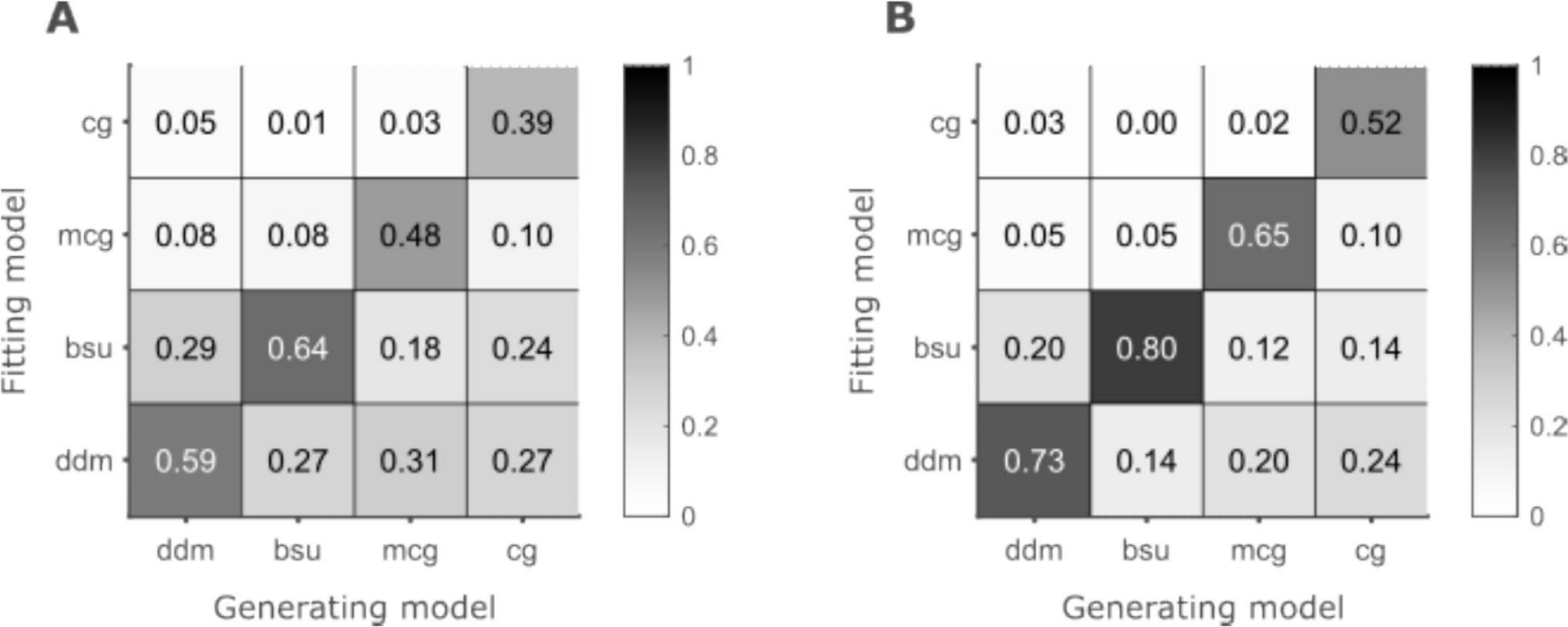
Model recoverability. **A** Recoverability using 225 trials, as in experiment 1. Each column shows the result of a single generating model, and hence sums to 1. Each row is for a single fitting model. **B** Same as **A** but for a larger experiment with 675 trials per participant.

After demonstrating the recoverability of the models’ parameters and exposing weaknesses when those were found, we tested to what extent it was possible to estimate which model generated a given dataset. We simulated the responses of 1,250 synthetic participants for each model using the same trial numbers as in experiment 1. We then fitted all four models to each of the resulting 5,000 simulations, and counted the number of simulations each model fitted best. A total of 225 trials, as we have in a single modality, yielded hit rates of 39% for CG, 48% for MCG, 59% for DDM, and 64% for BSU (Fig. 4). DDM and BSU, which are differentiated only by how they scale timing noise, were confused in 27–29% of the simulations. Increasing the number of trials by a factor of 3 to 675 trials improved the recoverability of all models as expected. The ordering of the models by hit rates remained the same: 52% for CG, 65% for MCG, 73% for DDM, and 80% for BSU.

To summarize the results of the simulations, we found that the parameters of the DDM and BSU were recovered well, while the boundary variability parameter of the CG and MCG was especially difficult to recover accurately. Model recoverability was also reasonable for the BSU and DDM using 225 trials per participant. The relatively low recoverability of MCG and CG is reflected by a tendency of DDM and BSU to fit data that they did not generate. This is another result of the low identifiability of the boundary variability parameter of MCG and CG. Despite having the same number of parameters, DDM and BSU have more functional flexibility than MCG and CG. We now turn to describe two experiments we conducted in order to test the models on empirical data.

## Experimental methods

The data and code for both experiments, as well as all analyses, are available at https://osf.io/87zbp/. “modality_script.m” contains the analysis of experiment 1, and “block_script.m” contains the analysis of experiment 2. “optimality_script.m” contains the code for the optimality analysis.

### Participants

A total of 85 individuals participated in two experiments. Forty participated in experiment 1 (32 female participants, average age = 23.9, standard deviation [*SD*] = 3) and 45 in experiment 2 (32 female participants, average age = 23.6, *SD* = 2.6). Six participants from each experiment were excluded from the analysis (15% and 13.3%, respectively), as they produced flat psychometric curves, meaning they were not responding to the intervals presented (Figs. S2 and S3).

## Experimental procedure

We report the results of two behavioral experiments, both run using OpenSesame (Mathôt et al., 2012). The first compared temporal generalization with visual versus auditory stimuli, and the second examined the effect of learning in the task. Both experiments used 400 ms as the standard duration and seven levels of stimulus duration as comparison stimuli (100, 200, 300, 400, 500, 700, and 800 ms).

In the first experiment, participants completed two parts, one containing visual stimuli and one containing auditory stimuli. The order of the parts was counterbalanced across participants. Each part included three blocks of 75 trials each, separated by breaks. The standard duration was presented five times at the start of each block. In total, all durations except the standard were presented 30 times and the standard 45 times in each modality. In other words, the standard appeared in 20% of trials. A white fixation dot appeared at the center of the screen whenever no stimuli were presented on the screen.

In the second experiment, we used visual stimuli in two levels of contrast (50% or 100%). The experiment contained six blocks of 80 trials each, separated by breaks. In addition to the trials, the standard duration was presented six times at the start of each block (three in each contrast). At the end of a block, the percent of accurate responses was presented on the screen. All durations except the standard were presented 60 times (30 in each contrast, or 20 in each block) and the standard 120 times (60 in each contrast, or 40 in each block). In other words, the standard appeared in 25% of all trials. Each block contained the same number of trials in each duration, displayed in a different order, to facilitate studying learning effects. A white fixation dot appeared at the center of the screen whenever no stimuli were presented on the screen.

In both experiments, participants could only respond once the stimulus was over. In previous experiments, feedback was provided after every trial (e.g., Wearden, 1992). We provided feedback at the end of every block of trials, by presenting the percentage of accurate responses during that block on the screen.

The experiments were not optimized for the collection of response times, which requires extra care when auditory stimuli are used. As a result, we only collected valid response times for experiment 2 and the visual part of experiment 1.

### Experiment 1 stimuli

Visual stimuli consisted of a square-wave grating presented in a circular window on a BenQ XL2420Z monitor running on 144 Hz, which was positioned 50 cm away from the participants. The grating had a spatial frequency of 1 cycle/cm and a diameter of 7 cm (corresponding to 8° visual angle) and was positioned at the center of the screen. Gratings were presented with a random orientation of 45° or 135°. Auditory stimuli were 500 Hz tones presented at a comfortable hearing level via Sennheiser HD 280 Pro headphones.

### Experiment 2 stimuli

Experiment 2 focused on the visual modality. The stimuli were the same square-wave gratings used in the visual part of experiment 1, presented in two contrast levels. Both contrast levels were used for standard and probe stimuli and varied randomly between trials. Previous studies report that higher-contrast stimuli are perceived as longer than lower-contrast counterparts (Matthews et al., 2011). Hence, we hypothesized that higher-contrast stimuli would shift the psychometric curve towards shorter intervals.

### Optimality analysis

We supplement our comparison of the different models on empirical data with an analysis of optimal behavior for the DDM. We defined a grid of 2,000 noise levels over the range we empirically found, excluding outliers as defined in the parameter recovery analysis. For each noise level, we searched for the decision boundaries that would maximize the probability of a correct response, or accuracy, given the same distribution of intervals as in experiment 1. We searched for accuracy-maximizing boundaries numerically using the Nelder–Mead algorithm, implemented in MATLAB’s fminserach() function. As explained in the results section, we repeated this analysis twice, with two duration distributions. First, we used the trial structure as in the true experiment, with 20% of trials containing the standard duration, and 13.33% for each of the other six durations. Second, we ran the analysis with 50% of trials containing the standard duration and 8.33% for each of the other durations. The curves outlining the optimal boundaries given different noise levels were then compared qualitatively to the actual parameter combinations found in our sample.

### Statistical analysis

Overall accuracy was analyzed using paired *t*-tests, implemented in MATLAB’s ttest() function. The probability of “same” response as a function of interval duration and other experimental manipulations was analyzed using analysis of variance (ANOVA) for repeated measures in JASP (version 0.19; JASP Team, 2024). We applied the Greenhouse–Geisser correction when Mauchly’s test indicated the data violated the sphericity assumption.

We analyzed the effect of experimental manipulation on cognitive parameters using two approaches. First, for both experiments, we followed the common approach of fitting a single model to the data for each participant in each condition separately, and then compared the parameters using *t*-tests or ANOVA. Second, for experiment 2, we complemented this analysis with a model comparison approach. A set of constrained models were fit to the data in addition to the full model. In each constrained mode, we kept one of the three parameters fixed across conditions while the rest were free to vary for each condition. This resulted in three constrained models: with fixed lower boundaries, fixed upper boundaries, or fixed diffusion coefficients. The models were compared at the group level. We computed Akaike’s information criteria for each model on the summed log-likelihoods and number of free parameters across participants.

Correlations between parameters and experimental conditions were tested using linear mixed models, implemented in MATLAB’s fitlme() function.

We used a bootstrap approach on the simulations from the parameter recovery analysis to create the null distribution of upper boundary and noise correlation. In each of 5,000 iterations, we drew as many synthetic participants as the same number of participants in the original experiment (33 and 36 for experiments 1 and 2, respectively) and calculated the Pearson correlation coefficient. The value observed in the empirical data was then compared to that distribution to compute a bootstrap *p* value.

## Experiment results

### The double-boundary DDM fits single participants’ data better than the other models

**Fig. 5:**
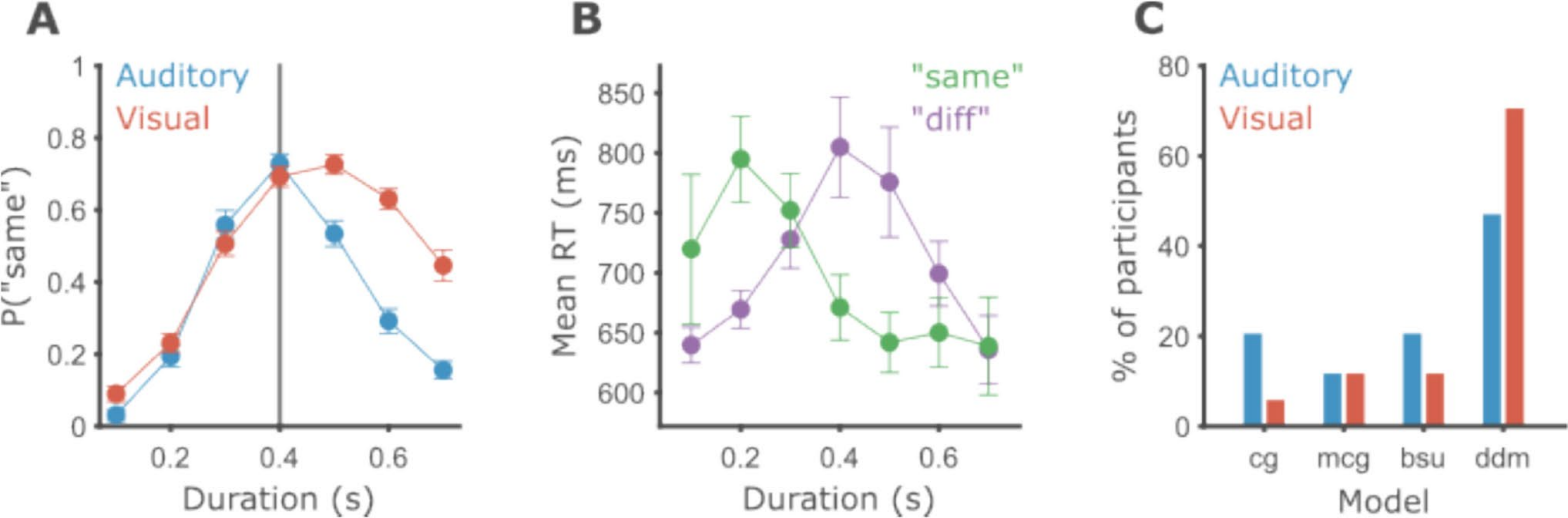
Participants perform better on auditory intervals. **A** Circles show the average probability of labeling an interval as “same” across participants, and error bars depict the within-participant standard error of the mean (SEM) using the Morey–Cousineau method (Cousineau et al., 2021). The vertical gray line corresponds to the standard duration. Blue indicates data from the auditory modality, and red indicates data from the visual modality. **B** Mean response times for “same” (green) and “different” (purple) responses by interval duration. Data from the visual modality only. **C** The percentage of participants (out of a total of 34) for which each model, at the *x*-axis, achieved the largest likelihood. Blue indicates data from the auditory modality, and red indicates data from the visual modality

We analyzed the data of 34 participants who completed two versions of temporal generalization, one block using auditory pure tones and one using visual gratings. Overall, participants were better with auditory stimuli (Fig. 5A). Participants performed significantly more accurately in the auditory modality (*M* = 71.01%, *SD* = 9.52%) than in the visual modality (*M* = 58.77%, *SD* = 8.88%). All participants but one had higher accuracy in the auditory modality (paired *t*-test, *t*(33) = 11.18, *p* < 0.001, *d* = 1.92).

We performed a repeated-measures ANOVA on the probability of “same” responses with interval duration (seven levels, 0.1–0.7 s), modality (two levels, audition or vision), and their interaction as within-participant factors. We found an expected significant main effect of interval duration, *F*(6, 33) = 92.23, *p* < 0.001, ${\eta }_{p}^{2}$ = 0.74, signifying that participants were attending to the task. In addition, we found a significant main effect of modality, *F*(1, 33) = 77.32, *p* < 0.001, ${\eta }_{p}^{2}$ = 0.7, as well as a significant duration by modality interaction, *F*(6, 33) = 17.23, *p* < 0.001, ${\eta }_{p}^{2}$ = 0.34.

Before exploring the differences between modalities in detail, we briefly describe the pattern of response times (RT) in the task. As the experiments were not optimized for the collection of RTs, we will describe only the results of the visual part (Fig. 5B). As expected, RT patterns differ depending on the decision. “Different” responses were quickest for the shortest and longest intervals and slowest around the standard duration. “Same” responses were slowest for short intervals, and plateaued at a quicker RT around the standard.

Visually inspecting participants’ binary performance shows that the psychometric curves for auditory and visual stimuli differ greatly in their shapes. The difference between modalities is most pronounced for longer intervals. Considering Weber’s law, this would intuitively correspond to a larger coefficient of variation in vision compared to audition: Timing noise for static gratings grows at a faster rate compared to pure tones. The models described in the first part of this work provide formal and statistical methods to test such intuitions, as explored below.

Comparing behavior under different conditions is often done by first summarizing behavior within conditions into model parameters and then comparing the parameter values between conditions. To do so, we need to establish whether the models are suitable, both in how well they can fit the data and in how well defined the model parameters are. First, we compared the ability of the four different models to fit data at a single participant level. For each participant, we fit each model to the data of each condition separately. Since all models have three free parameters, they can be directly compared in terms of their maximum likelihood. For both modalities, the DDM considerably outperformed all other models (Fig. 5C), with 47.1% and 73.5% of participants in the auditory and visual modalities respectively, compared with a chance level of 25% (Table 2). There was no obvious systematic difference between the other three models. Models BSU and CG were somewhat better on auditory than visual data. MCG performed the same in both modalities. Following previous research, we also fitted the models to the pooled data across participants. In the auditory modality, the original CG model performed best, while in the visual modality it was the MCG model. In both modalities, the DDM came in second.

**Table 2:** Comparison of model likelihoods in both modalities. Within each modality, the leftmost column includes the percentage and number of participants for which each model best fitted their data. The middle column includes the summed log-likelihood across participants as a measure of overall model fit. The right column includes the likelihood of the DDM divided by the likelihood of each model, summed across participants in log_10_ units. Since the number of free parameters is the same for all models, the likelihood ratio is equal to the ratio of Akaike weights (Wagenmakers & Farrell, 2004). Given the very large differences in likelihoods, measures of evidence weights, like Akaike weights, would give a weight of nearly 1 to the DDM and nearly 0 to the other three

	Audition			Vision		
	% (No.) of participants	Summed LL	Log_10_ (likelihood ratio)	% (No.) of participants	Summed LL	Log_10_ (likelihood ratio)
DDM	47.1 (16)	−3,279	0	70.6 (24)	−3,852	0
BSU	20.6 (7)	−3,364	37	11.8 (4)	−3,910	25
MCG	11.8 (4)	−3,675	172	11.8 (4)	−4,011	69
CG	20.6 (7)	−3,515	103	5.9 (2)	−4,188	146

As stated in the introduction, previous work on human participants only fit models to pooled data at the group level, which does not necessarily represent the behavior of single participants. Indeed, we found that despite being limited to capturing the behavior of single participants, the CG model provided the best fit for the group data in the auditory modality, and MCG provided the best fit in the visual modality. This result demonstrates that fitting to single participants is crucial when comparing models.

**Fig. 6:**
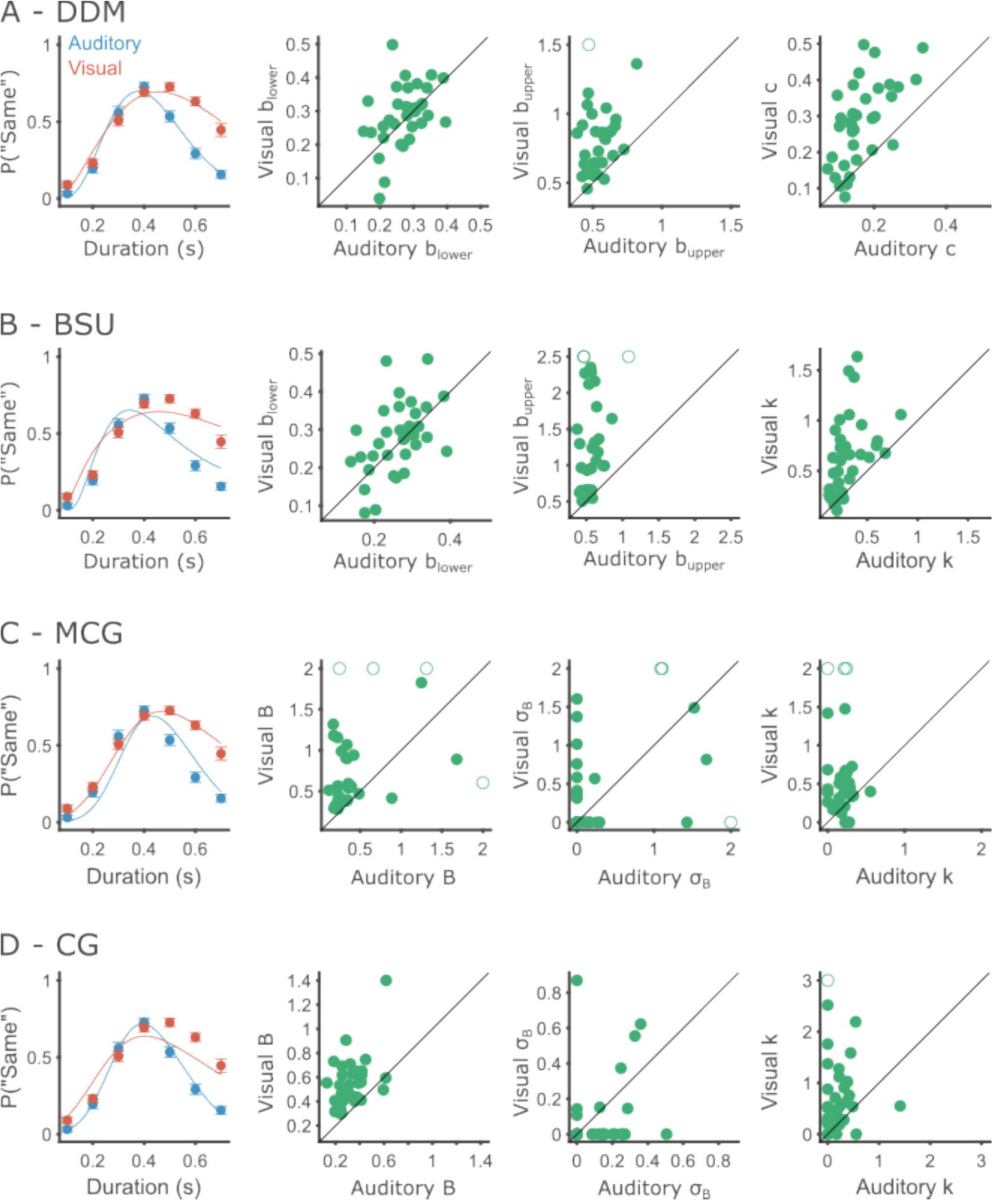
Behavioral performance and model fits for Experiment 1. Each row shows results of one model. **A** DDM. Leftmost is the performance and model fits at the group level. Circles show the average probability of labeling an interval as “same” across participants, and error bars depict the within-participant standard error of the mean (SEM). Scatter plots show the estimated values of each of the free parameters (Left to right: lower boundary, upper boundary and diffusion). X and Y axes correspond to the auditory and visual modalities, respectively. To facilitate visualization, parameters far from the group are truncated and depicted with empty circles. **B** Same as **A**, for the BSU model. Parameter scatter plots show, left to right, lower boundary, upper boundary, and Weber fraction. **C** Same as **A**, for the MCG model. Parameter scatter plots show, left to right: mean boundary, boundary standard deviation, and Weber fraction. **D** Same as **A**, for the CG model. Parameter scatter plots show, left to right, mean boundary, boundary standard deviation, and Weber fraction.

Both CG and MCG models have two parameters that control the slopes and asymmetry of the curves: boundary variability and memory variability, controlled by the Weber fraction. Having more than one parameter affecting the psychometric curve similarly could lead to identifiability problems, where changes to one parameter can be undone by changes to another parameter (Gershman, 2016; van Maanen & Miletić, 2021). This means that both parameters cannot be reliably estimated from typical empirical data at once. Identifiability is especially important if the parameters are used for inference, such as comparing between experimental conditions. To test parameter identifiability, we explored the estimated parameter values for all models (Fig. 6). Fits of both CG and MCG models show a tendency to shrink one of the two parameters towards zero, more often boundary variability, leaving the other parameter to absorb all explained variability (Table 3). This suggests that the slope parameters are unidentifiable. We note that this was already reported briefly by Wearden (1992). The CG model was only used to fit pooled data of several animals, each completing many hundreds of trials. These large amounts of data, atypical in human psychophysics, possibly allowed the fitting procedure to distinguish both sources of variability (Gibbon et al., 1984). However, for the type of data discussed here, these models are suboptimal.

**Table 3:** Parameter unidentifiability in the MCG and CG models. Each cell includes the number of participants (out of 34) for which the specific parameter (${\sigma }_{B}$, $k$, or both) was estimated to be smaller than 0.001

	MCG			CG		
	${\sigma }_{B}$	$k$	Both	${\sigma }_{B}$	$k$	Both
Audition	21	6	0	15	12	0
Vision	19	3	0	26	4	0

### Participants have less internal noise and use stricter decision boundaries when timing auditory vs. visual stimuli

Having established the DDM as a suitable model for analyzing single-participant data, we next used it to capture differences between conditions. We fit the DDM to the data for each participant within each condition separately (Fig. 6A) and compared the estimated parameters between the conditions using paired *t*-tests. Lower boundaries were not significantly different between modalities (*M*_aud_ = 0.27, *SD*_aud_ = 0.06, *M*_vis_ = 0.29, *SD*_vis_ = 0.09; *t*(33) = 0.99, *p* = 0.329, *d* = 0.17). In contrast, both the diffusion and upper boundaries were strongly affected by the modality. Diffusion coefficients were significantly larger in the visual condition (*M*_aud_ = 0.17, *SD*_aud_ = 0.06, *M*_vis_ = 0.28, *SD*_vis_ = 0.11; *t*(33) = 7.50, *p* < 0.001, *d* = 0.77), and participants placed their upper boundaries at longer intervals in the visual condition (*M*_aud_ = 0.54, *SD*_aud_ = 0.09, *M*_vis_ = 0.83, *SD*_vis_ = 0.38; *t*(33) = −4.51, *p* < 0.001).

As explained in the parameter recovery analysis, upper boundaries larger than roughly 0.8 tend to be overestimated. In our data, estimated upper boundaries above 0.8 s occurred only once in the auditory condition, but 17 times (50% of participants) in the visual condition. This reveals a limitation in the experimental design. It is possible that the range of intervals used in the experiment was too difficult for many of our participants in the visual modality. Yet, the effect we found is robust despite this limitation, since all participants but two had larger upper boundaries in the visual modality.

The fact that both internal noise and upper boundary were significantly different between modalities motivated us to explore whether both parameters might be inherently related. If this were the case, both parameters should be correlated across participants. To make sure our statistical analysis was not overly affected by outlier values, we checked for upper boundaries or diffusion coefficients that were 3.5 standard deviations or more away from the respective means. We excluded one participant with an upper boundary of 2.6, which is 6.27 standard deviations above the mean upper boundary. We ran a linear mixed effects model predicting the upper boundary using modality (binary predictor with effects coding: −1 for audition and 1 for vision) and diffusion (continuous predictor) as fixed effects. The intercept and slope against diffusion were set as random effects.

**Fig. 7:**
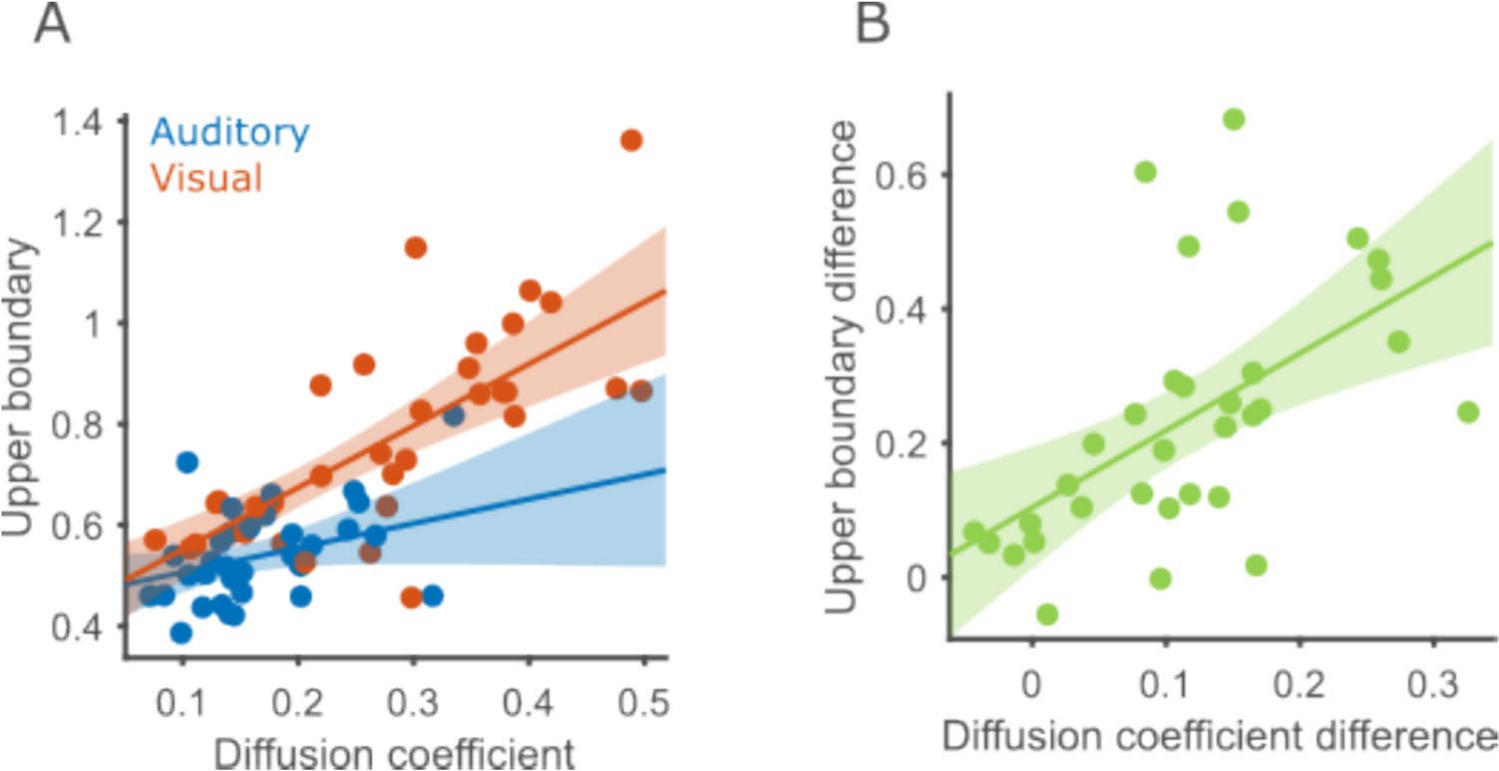
Diffusion coefficient and upper boundary are correlated. **A** Correlation of diffusion coefficient and upper boundary in both modalities. Circles represent the data of a single participant in a single modality (blue for auditory and red for visual). The 95% confidence interval of the regression lines are marked using shaded ribbons. **B** Correlation across participants between upper boundary difference (vision − audition) and diffusion coefficient difference.

We found that larger diffusion coefficients correlated with larger upper boundaries (β = 0.91, *p* < 0.001; Fig. 7A). Modality still predicted significant variability in upper boundaries, even after taking diffusion into account (β = −0.07, *p* < 0.001). Additionally, the relation between diffusion and upper boundary was stronger in the visual modality, as indicated by a significant interaction of modality and diffusion (β = −0.38, *p* = 0.011). Importantly, the correlations seen in the empirical data are larger than those seen in the parameter recovery analysis. The Pearson correlation between upper boundary and diffusion was estimated at 0.775. The probability of finding a correlation equal to or larger than that in the simulations from the parameter recovery analysis was 0.01. Hence, this correlation reveals a true link between the two measures, rather than an artifact of the fitting procedure or model specification. If diffusion and upper boundaries are related, another prediction is that participants who displayed a larger effect of modality on their diffusion coefficient should also display a larger effect of modality on upper boundaries. Hence, we computed the difference in the diffusion coefficient between modalities (vision minus audition, so we generally expect positive differences) as well as the difference of upper boundaries and correlated the two. The two differences were significantly correlated (Pearson ρ = 0.56, *p* < 0.001; Fig. 7B), in line with the prediction. In summary, upper boundaries and diffusion are related, yet the effect of modality on upper boundaries is not completely explained by its effect on internal noise.

### Psychophysical functions become narrower with increased experience in the task

We analyzed the data of 39 participants in the second experiment, exploring the effect of learning in the task. We performed a repeated-measures ANOVA on the probability of “same” responses with interval duration (seven levels, 0.1–0.7 s), block (three levels, blocks 1–2, 3–4, and 5–6), contrast (two levels, 50% and 100%), and all interactions as within-participant factors. As expected, there was a significant main effect of interval duration, *F*(2.4, 91.06) = 133.81, *p* < 0.001, ${\eta }_{p}^{2}$ = 0.78. More importantly, we found a significant main effect of block number, *F*(1.71, 65.06) = 18.58, *p* < 0.001, ${\eta }_{p}^{2}$ = 0.33, as well as a significant duration by block interaction, *F*(4.642, 176.41) = 5.84, *p* < 0.001, ${\eta }_{p}^{2}$ = 0.13. Contrast, block by contrast, duration by contrast, and duration by block by contrast were not significant, *F*(1, 38) = 0.06, *p* = 0.815, ${\eta }_{p}^{2}$ = 0.001; *F*(2, 76) = 0.43, *p* = 0.655, ${\eta }_{p}^{2}$ = 0.011; *F*(4.18, 158.87) = 2.189, *p* = 0.07, ${\eta }_{p}^{2}$ = 0.054; *F*(7.84, 297.87) = 1.33, *p* = 0.232, ${\eta }_{p}^{2}$ = 0.034, respectively.

**Fig. 8:**
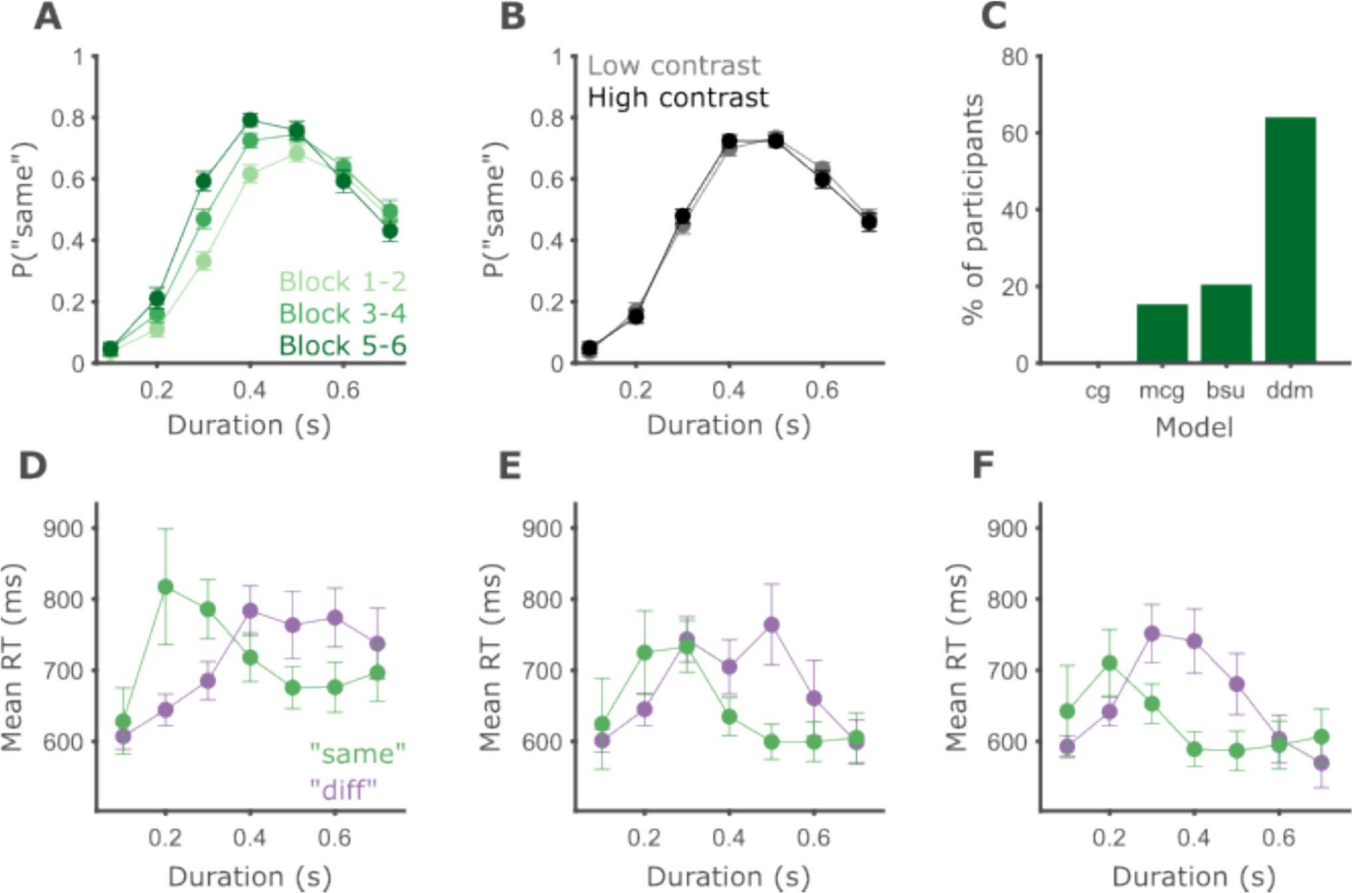
Psychophysical performance improves with learning. **A** Circles show the average probability of labeling an interval as “same” across participants, and error bars depict the within-participant standard error of the mean (SEM). Darker color corresponds to later blocks in the experiment. **B** Same as **A**, except that trials are separated into two groups by the contrast of the visual grating. **C** The percent of participants (out of a total of 39) for which each model, at the *x*-axis, achieved the largest likelihood. **D–F** Mean response times for “same” (green) and “different” (purple) responses by interval duration in the blocks 1–2, 3–4, and 5–6, respectively.

The significant duration by block interaction means participants systematically modified their behavior over the course of the experiment. Visually inspecting participants’ performance shows that the generalization gradients became narrower with longer experience with the task and increasing exposure to the standard durations (Fig. 8A). To explore the cognitive underpinnings of this change with learning, we turn to modeling the behavior.

First, we compared the models in terms of their ability to fit the data. As shown in Table 4, the DDM provided the best fit for 64.1% of the participants, followed by the BSU with 20.5%, MCG with 15.4%, and CG which did not fit any participant best (Fig. 8C). For the rest of the analysis, we focus on the DDM.

**Table 4:** Comparison of model likelihoods. The likelihoods are compared between participants across blocks. The leftmost column includes the percentage and number of participants for which each model best fitted their data. The middle column includes the summed log-likelihood across participants, as a measure of overall model fit. The right column includes the likelihood ratio of the DDM against each model, in log_10_ units

	% (No.) of participants	Summed log-likelihood	Log_10_ (likelihood ratio)
DDM	64.1 (25)	−8,646	0
BSU	20.5 (8)	−8,763	50
MCG	15.4 (6)	−9,125	208
CG	0 (0)	−9,939	561

**Fig. 9:**
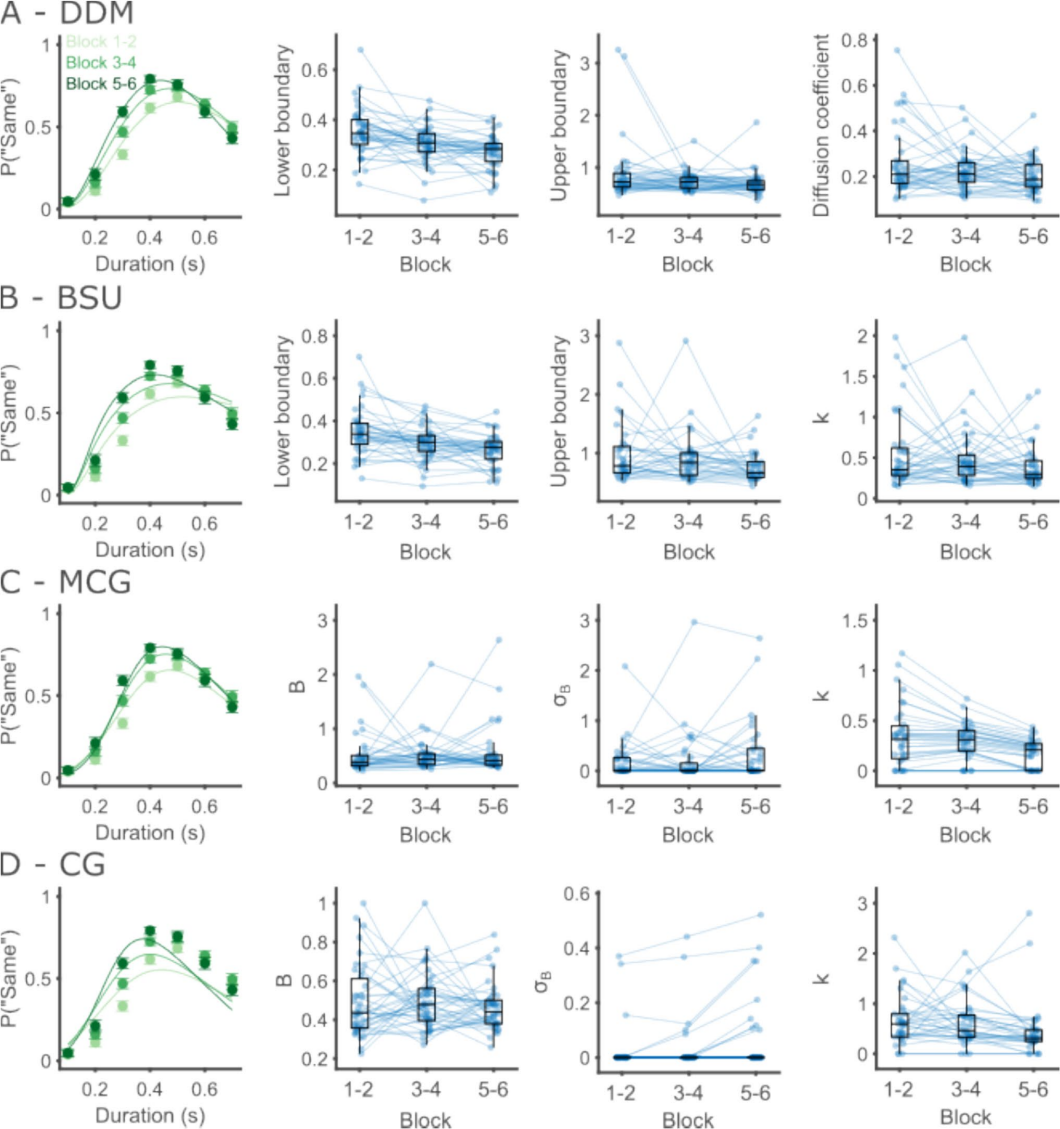
Psychophysical performance improves with learning. **A** Group fits and individual parameter estimates for each block using the DDM. Left, performance and model fits at the group level. Circles show the average probability of labeling an interval as “same” across participants, and error bars depict the within-participant standard error of the mean (SEM). Scatter plots show the estimated values of each of the free parameters for data of each block. Circles and connecting lines mark the parameter values across blocks for a single participant, with box plots overlaid. Horizontal lines within the boxes represent the group median, the box extends from the 25th percentile to the 75th percentile, and the whiskers extend 1.5 IQRs from the median. **B** Same as **A** but for the BSU model. For visualization clarity, participants with lower boundaries above 0.8, upper boundaries above 3, and Weber fraction above 2 in any of the blocks are not shown. **C** Same as **A** but for the MCG model. For visualization clarity, participants with mean boundary separation above 3, boundaries standard deviation above 3, and Weber fraction above 1.5 in any of the blocks are not shown. **D** Same as **A** but for the CG model. For visualization clarity, participants with boundaries standard deviation above 0.55 and Weber fraction above 3 in any of the blocks are not shown.

We then performed a repeated-measures ANOVA on the estimated parameters with block (three levels, blocks 1–2, 3–4, and 5–6) as a within-participant factor. We found that the lower boundary differed significantly between blocks, *F*(2, 76) = 14.82, *p* < 0.001, ${\eta }_{p}^{2}$ = 0.28, shifting to shorter durations with learning (Tukey–Kramer post hoc tests, first vs. second tertile: *p* = 0.010, first vs. third: *p* < 0.001, second vs. third: *p* = 0.021). Diffusion coefficients also changed significantly between blocks, *F*(2, 76) = 4.70, *p* = 0.012, ${\eta }_{p}^{2}$ = 0.11, becoming somewhat smaller over the course of the experiment. A post hoc test found a significant difference between the first and third tertiles (first vs. second tertile: *p* = 0.275, first vs. third: *p* = 0.017, second vs. third: *p* = 0.237). Upper boundaries did not differ significantly between blocks, *F*(2,76) = 2.33, *p* = 0.105, ${\eta }_{p}^{2}$ = 0.06. Given that upper boundaries remain quite close to the edge of the interval range even at the last two blocks of the experiment, it is possible that using a wider range of intervals would uncover systematic changes in the upper boundaries as well (Fig. 9).

We complement this analysis with a model comparison approach, constraining each parameter to have a fixed value across blocks in turn. Ordering the models by their AIC, the best model was the full DDM in which all parameters were free to vary between blocks (Table 5). The full model was followed by a model with constrained noise levels, then a model with constrained upper boundaries, and finally by a model with constrained lower boundaries. The model comparison and parameter comparison approaches agree on the clear differences in lower boundaries between blocks. They disagree on whether upper boundaries differ between blocks. While the parameter ANOVA did not find a significant difference in upper boundaries, AIC prefers a model in which both boundaries vary but noise remains the same over a model in which noise and lower boundaries vary but upper boundaries remain the same. This could result from the few participants with very large differences in their upper boundaries. While these datapoints would result in large estimates of within-block variability in the ANOVA, which goes against the between-block variability, their effects only add up in the model comparison approach we used.

**Table 5:** Model comparison results. AIC values are compared against the full DDM

	Full	Constrained noise	Constrained lower boundaries	Constrained upper boundaries
ΔAIC	0	490	252	97

**Fig. 10:**
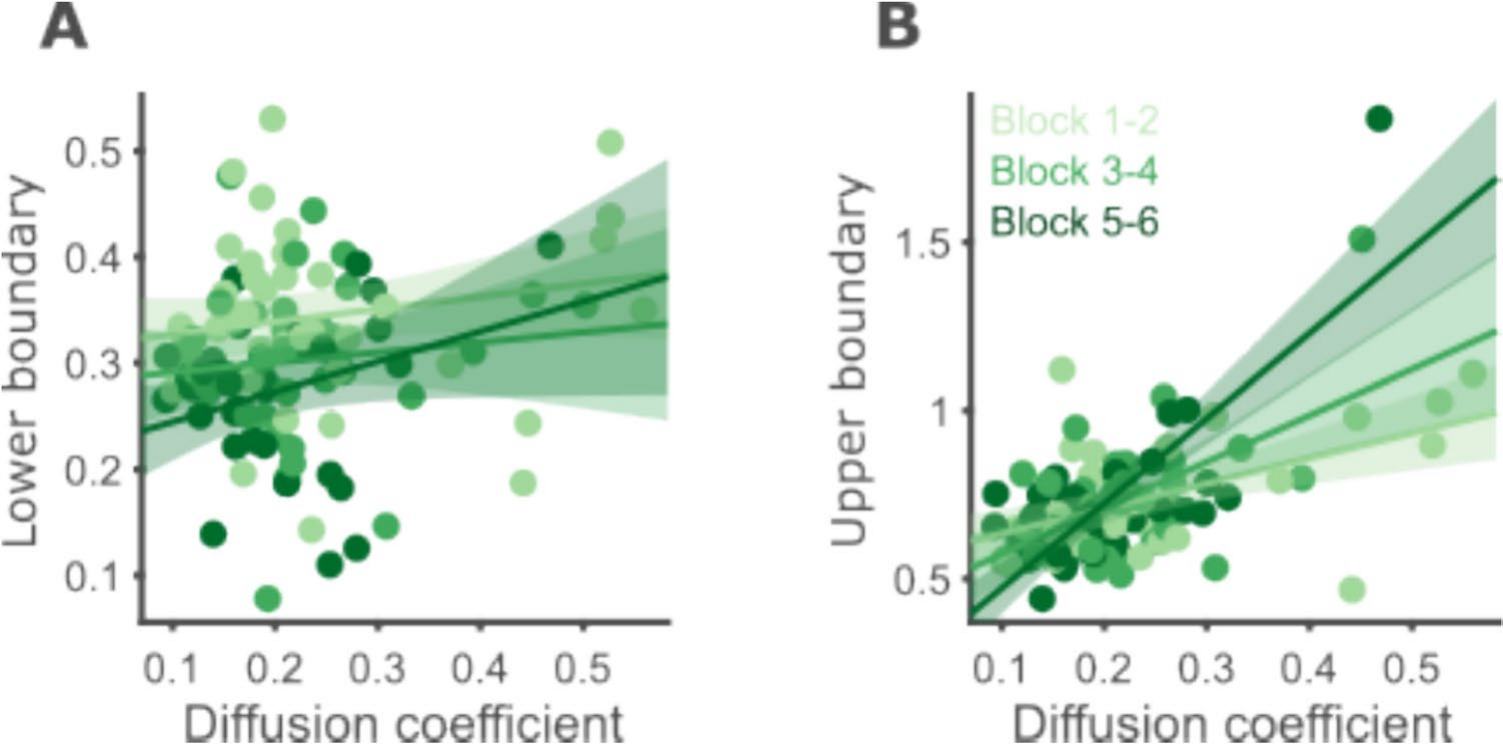
Diffusion coefficients are correlated with upper, but not lower, boundaries. **A** Correlation of diffusion coefficient and lower boundary in the three tertiles. Each circle represents the data of a single participant in a single tertile (darker colors for later tertiles). The 95% confidence intervals of the regression lines are marked using shaded ribbons. **B** Same as **A**, but for upper boundaries. For visualization, we used the same linear model as for lower boundaries.

Here, as in the comparison between modalities, we found two parameters of the model—diffusion coefficient and lower boundary—that changed significantly between blocks. As before, we first tested for outlier values in lower boundaries and diffusion coefficients, with 3.5 standard deviations away from the mean as the rejection criterion. We removed one participant who had a diffusion coefficient in the first block 4.8 standard deviations above the mean. We next used a linear mixed effects model to predict the lower boundary using block (categorical predictor with the first block as the reference level), diffusion coefficient (continuous predictor) and the diffusion coefficient by block interaction as fixed effects and a random intercept per participant. As expected, lower boundaries were gradually shifted to shorter intervals over blocks (second vs. first tertile, β = −0.04, *p* = 0.003; third vs. first tertile, β = −0.06, *p* < 0.001). The diffusion coefficient was not significantly correlated with lower boundaries in the first tertile (β = 0.12, *p* = 0.165). The interaction terms were also not significant, suggesting similar relations of diffusion to lower boundaries over learning (second vs. first tertile, β = −0.02, *p* = 0.864; third vs. first tertile, β = 0.16, *p* = 0.284; Fig. 10A). The mixed model results suggest that learning affects the lower boundary and diffusion coefficient independently.

We next tested the relation between upper boundaries and noise to corroborate the results of experiment 1. For this analysis, we removed two participants whose upper boundaries, and one participant whose diffusion coefficient, were farther than 3.5 SDs from the mean. Across blocks, upper boundaries were positively correlated with diffusion, as in experiment 1 (rho = 0.56, *p* < 0.001). A correlation as large or larger occurred in the parameter recovery simulations at a probability of 0.04.

### Participants’ boundaries are close to optimal given their levels of noise under the assumption of equal stimulus proportions

In both experiments, we found that upper boundaries and diffusion coefficients are strongly correlated, while lower boundaries and diffusion are not. The correlation between the parameters in real data was much larger than that observed in the parameter recovery analysis. This suggests that the correlation we found reflects a cognitive constraint or strategy. From a cognitive perspective, while boundaries are likely to be under some level of control, noise levels are typically considered as something that cannot be “willfully” modulated, representing a limiting factor on performance (Carandini, 2024; Gardner, 2019). In our experimental design, no direct reward is given, feedback on overall accuracy is provided at the end of each block of trials, and no response deadlines are used. Hence, we consider optimality in terms of accuracy, rather than reward rates.

**Fig. 11:**
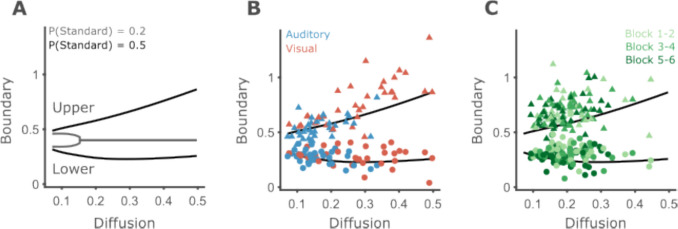
Participants’ boundaries are close to optimal under the assumption of equal stimulus proportions. **A** Upper and lower boundaries for different levels of noise, assuming *P*(standard) = 0.2 (gray) or *P*(standard) = 0.5 (black). For noise levels larger than about 0.15 and *P*(standard) = 0.2, the accuracy-maximizing strategy is to always respond “different,” implemented by placing both boundaries at 0.4. **B** Observed boundaries (upper and lower boundaries as triangles and circles, respectively) vs. accuracy-maximizing boundaries (black lines), assuming that *P*(standard) = 0.5. Blue symbols correspond to data from the auditory part of experiment 1 and red correspond to data from the visual part. **C** Same as **B**, but for experiment 2. Light green for the first two blocks of trials, and dark green for the last two blocks.

We note that the probabilities of standard and nonstandard durations are unbalanced in both experiments. In experiment 1, the probability of a standard duration is 0.2, and in experiment 2 it is 0.25. Under such conditions, the optimal strategy in terms of accuracy becomes always guessing “different” for relatively low levels of noise (around 0.15, Fig. 11A). In the DDM, this strategy is implemented by placing the boundaries at the same value, such that the probability of the accumulator being between the boundaries is 0. However, many of our participants displayed levels of noise considerably larger than 0.15, and no participant always replied “different.”

A possible explanation is that participants assume that standard and nonstandard durations occur equally often, as they cannot know the true proportions. Assuming *P*(standard) = 0.5, participants’ fitted boundaries generally match what is predicted for their level of noise (Fig. 11B, C). An exception is seen for high upper boundaries, which were found to often fall above the predicted line. However, as noted in the parameter recovery section, upper boundaries close or beyond the maximal duration used (0.7 s) tend to be overestimated.

## Discussion

A diverse set of neural mechanisms is thought to underlie our ability to track the passage of time (Paton & Buonomano, 2018). Yet, at the algorithmic level, many timing behaviors can be described as bounded accumulation processes (Balcı & Simen, 2024). The drift-diffusion framework, which incorporates time perception into the wider perceptual decision-making literature, has been applied to a range of timing behaviors. DDMs are a natural framework to explain binary responses as well as response times in temporal bisection (Balcı & Simen, 2014), and can also account for EEG patterns in this task (Ofir & Landau, 2022, 2025). In this work, we further extend the framework to temporal generalization.

The previous models and the proposed DDM all share the basic design of psychophysical models, that is, comparing a decision variable against decision boundaries, but vary considerably in their assumptions regarding the boundaries and the sources of variability. The original models, CG and MCG, constrained decision boundaries to be symmetric around the true standard duration and assumed that performance variability in the task (i.e., the slopes of psychophysical functions) is driven by noise in the memory of the standard and the decision boundaries. A more recent model, BSU, relaxed the constraint on the decision boundaries, and removed their trial-to-trial variability. Here, we apply the drift-diffusion approach to temporal generalization. We defined two decision boundaries, free to take any values, and a diffusion coefficient controlling the level of internal noise.

We found that the DDM had the most recoverable parameters, with low parameter trade-offs. BSU achieved close, if somewhat weaker, parameter recoverability. The analysis revealed significant difficulty in estimating the decisional variability parameter of the MCG and CG models. We also found that model recoverability was not uniform across models, with the BSU leading and the DDM behind it. Increasing the number of trials improved the recoverability of all models. The results of the parameter and model recovery analyses can be thought of as tests of the models’ efficiency: Given a specific number and design of trials, how well can we estimate the model parameters? Such questions have been explored before for sigmoid psychometric curves (e.g., Wichmann & Hill, 2001), but not for non-monotonic curves, such as in temporal generalization.

In addition, the DDM outperforms all other models in fitting single participants’ data. This is found for timing using both auditory stimuli and visual stimuli. Fitting to empirical data also made clear a trade-off between the two noise parameters of the CG and MCG models. For most participants, one of the parameters was estimated at zero, leaving all variance to be explained by the other. This does not mean that there is no variability in one of the components (decision or timing). However, without very large datasets for single participants, it is not possible to distinguish the two sources of variability.

The better performance of the DDM and BSU models compared to the MCG and CG models likely reflects a more efficient use of available degrees of freedom. While MCG and CG use two parameters for modeling distinct sources of noise, DDM and BSU use two parameters for fitting shifted psychometric curves. Empirically, shifted curves are prevalent and cannot be properly fitted with models that assume symmetric boundaries around the true standard. On the other hand, a single source of noise is apparently enough to provide a satisfactory fit of behavior in typical experiments. The distinction between DDM, the leading model, and BSU is only in how noise scales with time. While in the DDM the standard deviation of the decision variable grows with the square root of time, BSU assumes linear growth. This is surprising, as models of timing often assume that the standard deviation of perceived duration grows linearly with time (Hass & Durstewitz, 2016, but see Scott et al., 2015; Simen et al., 2011). DDMs offer a unique perspective here: The standard deviation of the decision variable position grows with the square root of time, while the standard deviation of the first-passage time grows linearly. The original timing DDMs focused on first-passage times, and here we focus on the variability of the decision variable, disregarding when it crosses a boundary. Future studies could explore this distinction in more detail.

Having a model which can be robustly fitted to single participants’ data enables the statistical testing of how experimental manipulations affect the cognitive processes underlying behavior. Using the proposed DDM, we corroborated the well-documented finding that auditory timing is better than visual timing. We extend that finding by showing that the larger timing noise of visual stimuli is accompanied by a strategic adaptation at the decision-making level. Furthermore, participants with larger timing noise also had higher upper boundaries, in both modalities. The correlation between timing noise and the upper boundary can be explained as an accuracy-maximizing strategy. This is in line with previous research, which indicates that participants adapt their behavior according to their level of internal noise (Freestone & Church, 2016; Jazayeri & Shadlen, 2010; Kononowicz et al., 2022; Maaß et al., 2021; Simen et al., 2011). Assuming that noise is not modifiable by the participant, but that boundary placement is, suggests a causal direction: Modality affects timing noise, and timing noise affects behavioral strategy.

We additionally used the DDM to uncover the cognitive processes affected by learning in the task, using data from a second temporal generalization experiment. At the raw behavioral level, we found that psychophysical curves became narrower and shifted to shorter intervals with increased experience with the task. These changes are captured in the DDM as a lowering of the lower decision boundaries, accompanied by a decrease in the diffusion coefficient. We found that participants initially placed the lower boundary almost at the true standard duration. As they gained practice with the task, participants gradually shifted their lower boundaries to shorter durations, which might reflect a more accurate representation of the standard.

Our findings contrast with a previous study which suggested that upper, but not lower, boundaries are lowered with learning (Wearden & Towse, 1994). However, that study, which relied on the MCG model and a variant of it, did not statistically compare parameter estimates between blocks of trials, and only analyzed the pooled data of all participants, which can be misleading. Additionally, their pooled behavioral results (Wearden & Towse, 1994; Fig. 2) reflect overall better psychophysical performance than what we find. For instance, their participants categorized a 700 ms interval as “same” in less than 20% of the trials, while in our sample the pooled data yield a value of about 50%. This might be expected, as Wearden and Towse provided feedback after every response, while we provided feedback only at the end of each block, in the form of percentage of accurate responses. Per-trial feedback is probably especially important in the longer part of the range, where participants are expected to have low confidence in their decisions owing to Weber’s law. These differences make direct comparisons difficult.

It is not clear why participants placed their lower boundaries so close to the standard at the beginning of the task. Assuming that participants stop monitoring the interval when they have made their decision (but see Kononowicz & Van Rijn, 2014), one could speculate that participants start with high decision boundaries as a way to ensure that all available information is accumulated. As participants gain more knowledge about the distribution of the intervals in the task, they shift their decision boundaries to shorter intervals, which enables earlier preparation of responses and potentially increases the reward rate (Balci et al., 2011; Masís et al., 2023). Similarly, when participants are instructed to respond as quickly as possible in temporal generalization, even before the interval is over, the entire psychometric curve is shifted towards shorter intervals (Klapproth & Müller, 2008; Klapproth & Wearden, 2011). Critically, in those experiments, early responding terminated the intervals, thus giving participants control over the distribution of intervals they encounter. In our experiments, participants could not respond before intervals’ offset, which would argue against that explanation in the experiment we report here.

We also explored the relation between parameters through an optimality framework. We find that participants place their boundaries as would be expected given their noise levels, assuming that they respond as if standards are equally likely as non-standards. The optimality analysis also explains the lack of correlation between the noise level and the lower boundary. The optimal placement of the lower boundary does not depend on noise to the same extent as for upper boundaries. Compared to the large variability of upper boundaries for different levels of noise, lower boundaries vary only slightly.

Intuitively, we would expect participants to learn through experience in the task that the standard duration appears less frequently than 50% of trials. This assumption means that over blocks, lower boundaries would shift to longer durations while upper boundaries would decrease towards shorter durations. This contrasts with what we find: a decrease in lower boundaries, with nonsignificant differences in the upper boundaries. Future studies can explore exactly how the probability of a standard is learned over experience in the task.

Looking forward, there are some elements of our model which deserve additional scrutiny. First, we chose to parameterize our DDM using the decision boundaries rather than an internal representation of the standard duration. However, the boundaries themselves presumably only demarcate a “confidence interval” around the standard duration. An alternative parameterization could instead describe an internal reference with symmetric boundaries around it. Such a parameterization would yield identical fits to the one we use, but could be worthwhile from a conceptual perspective. For example, it emphasizes the fact that all four models considered here assume that decision boundaries, or the representations of the standard, are stable across trials (DDM and BSU) or vary randomly from trial to trial (CG and MCG). Past studies about time perception as well as many other forms of perception show that psychophysical behavior is strongly correlated between trials, a phenomenon known as serial dependence (Cicchini et al., 2024; Hachen et al., 2021; Raviv et al., 2012; Taatgen & van Rijn, 2011; Wiener & Thompson, 2015). Serial dependence could be leveraged to test whether upper and lower decision boundaries change together or independently across trials. If they change together, this could favor a parameterization which includes the standard explicitly, rather than the current DDM parameterization which treats the boundaries as independent from each other.

Finally, in the proposed model we assumed that the decision boundaries only apply at the offset of the interval. The decision variable is free to cross the boundaries during the interval without triggering a decision. Intuitively, this assumption is probably false, as is easiest to see in trials in which the interval is long enough, such that the upper boundary has been reached before the interval offset. In those cases, a “different” decision can be committed to before the interval is over. Previous research found that when participants are only able to respond after a go cue, instead of as soon as they make their decision, they do not integrate all available information. Instead, participants only accumulate evidence until they reach a decision boundary, regardless of when the evidence stops (Kiani et al., 2008). In time perception, such an approach is all the more reasonable, as time—and hence evidence—only evolves in one direction. In other words, relatively long intervals could be categorized as “different” before their offset, as soon as the decision variable reaches the upper boundary.

Two predictions can be made based on this hypothesis, at the behavioral and physiological levels. First, the fact that for longer intervals “different” decisions can be made before interval offsets predicts faster responses in those cases, as motor preparation can start in advance. We do not see clear evidence for that in our data, but our experiment was not optimized for the collection of RTs. Previous studies provide some evidence that this is the case in temporal generalization (Klapproth, 2018; Klapproth & Müller, 2008; Klapproth & Wearden, 2011). This response speed-up is widely reported in temporal bisection, where “long” decisions are often committed before interval offset (Balcı & Simen, 2014; Wiener et al., 2014; Wiener & Thompson, 2015).

Second, breaking the decision process into two stages, until interval offset and after it, suggests that we can expect to see a similar pattern of neural signatures in temporal generalization and bisection. Specifically, it is expected that the offset-evoked potential in temporal generalization will be larger for shorter intervals, as it is in bisection (Ofir & Landau, 2022), and that motor preparation will be apparent before offset (Ofir & Landau, 2025). To our knowledge, only two studies have examined the offset-evoked potential in temporal generalization (Bannier et al., 2019; Özoğlu & Thomaschke, 2023). Both studies found the same pattern of nonlinear decreasing EEG potential as a function of interval duration in temporal generalization and bisection. A two-stage DDM, like the one developed for temporal bisection, would be a beneficial tool for future studies recording response times alongside noninvasive physiology to study the temporal dynamics of the decisions in the temporal generalization task.

In summary, we provide a model for temporal generalization which can robustly fit single-participant data. This enables us to directly test the effect of stimulus modality and learning on the cognitive processes that are involved in temporal generalization. We hope this advance in the behavioral analysis of temporal generalization will facilitate future exploration of this task, both at the behavioral level and as a basis for relating neural activity to the underlying cognitive processes.

## Supplementary Information

Below is the link to the electronic supplementary material.

Supplementary file1 (DOCX 565 KB)

## Data Availability

The data and code for all experiments are available at https://osf.io/87zbp/.

## Code availability

The code for all analyses is available at https://osf.io/87zbp/.

## Declarations

### Open Practices Statement

The data and code for all experiments and analyses are available at https://osf.io/87zbp/. Neither of the experiments was preregistered.

## References

[CR1] Allan, L. G., & Gibbon, J. (1991). Human bisection at the geometric mean. *Learning and Motivation,**22*(1–2), 39–58. 10.1016/0023-9690(91)90016-2

[CR2] Balci, F., Simen, P., Niyogi, R., Saxe, A., Hughes, J. A., Holmes, P., & Cohen, J. D. (2011). Acquisition of decision making criteria: Reward rate ultimately beats accuracy. *Attention, Perception, & Psychophysics,**73*(2), 640–657. 10.3758/s13414-010-0049-7 10.3758/s13414-010-0049-7 PMC3383845 21264716

[CR3] Balcı, F., & Simen, P. (2014). Decision processes in temporal discrimination. *Acta Psychologica,**149*, 157–168. 10.1016/j.actpsy.2014.03.005 24726447 10.1016/j.actpsy.2014.03.005

[CR4] Balcı, F., & Simen, P. (2016). A decision model of timing. *Current Opinion in Behavioral Sciences,**8*, 94–101. 10.1016/J.COBEHA.2016.02.002

[CR5] Balcı, F., & Simen, P. (2024). Neurocomputational Models of Interval Timing: Seeing the Forest for the Trees. In H. Merchant & V. De Lafuente (Eds.), *Neurobiology of Interval Timing* (Vol. 1455, pp. 51–78). Springer International Publishing. 10.1007/978-3-031-60183-5_4 10.1007/978-3-031-60183-5_4 38918346

[CR6] Bannier, D., Wearden, J., Le Dantec, C. C., & Rebaï, M. (2019). Differences in the temporal processing between identification and categorization of durations: A behavioral and ERP study. *Behavioural Brain Research,**356*, 197–203. 10.1016/j.bbr.2018.08.027 30189287 10.1016/j.bbr.2018.08.027

[CR7] Bausenhart, K. M., Di Luca, M., & Ulrich, R. (2018). Assessing Duration Discrimination: Psychophysical Methods and Psychometric Function Analysis. In A. Vatakis, F. Balcı, M. Di Luca, & Á. Correa (Eds.), *Timing and Time Perception: Procedures, Measures, & Applications*. BRILL. 10.1163/9789004280205

[CR8] Birngruber, T., Schröter, H., & Ulrich, R. (2014). Duration perception of visual and auditory oddball stimuli: Does judgment task modulate the temporal oddball effect? *Attention, Perception, & Psychophysics,**76*(3), 814–828. 10.3758/s13414-013-0602-2 10.3758/s13414-013-0602-2 24435899

[CR9] Carandini, M. (2024). Sensory choices as logistic classification. *Neuron,**112*(17), 2854-2868.e1. 10.1016/j.neuron.2024.06.016 39013468 10.1016/j.neuron.2024.06.016 PMC11377159

[CR10] Church, R. M., & Deluty, M. (1977). Bisection of temporal intervals. *Journal Of Experimental Psychology: Animal Behavior Processes,**3*(3), 216–228. 881613 10.1037//0097-7403.3.3.216

[CR11] Church, R. M., & Gibbon, J. (1982). Temporal generalization. *Journal of Experimental Psychology: Animal Behavior Processes,**8*(2), 165–186. 10.1037/0097-7403.8.2.165 7069377

[CR12] Cicchini, G. M., Arrighi, R., Cecchetti, L., Giusti, M., & Burr, D. C. (2012). Optimal encoding of interval timing in expert percussionists. *The Journal of Neuroscience,**32*(3), 1056–1060. 10.1523/JNEUROSCI.3411-11.2012 22262903 10.1523/JNEUROSCI.3411-11.2012 PMC6621155

[CR13] Cicchini, G. M., Mikellidou, K., & Burr, D. C. (2024). Serial dependence in perception. *Annual Review of Psychology,**75*, 129–154. 10.1146/annurev-psych-021523-104939 37758238 10.1146/annurev-psych-021523-104939

[CR14] Cousineau, D., Goulet, M.-A., & Harding, B. (2021). Summary Plots With Adjusted Error Bars: The *superb* Framework With an Implementation in R. *Advances in Methods and Practices in Psychological Science,**4*(3), 251524592110351. 10.1177/25152459211035109

[CR15] Di Luca, M., & Rhodes, D. (2016). Optimal perceived timing: Integrating sensory information with dynamically updated expectations. *Scientific Reports,**6*(1), 28563. 10.1038/srep28563 27385184 10.1038/srep28563 PMC4935895

[CR16] Droit-Volet, S., Clément, A., & Wearden, J. (2001). Temporal generalization in 3- to 8-year-old children. *Journal of Experimental Child Psychology,**80*(3), 271–288. 10.1006/jecp.2001.2629 11583526 10.1006/jecp.2001.2629

[CR17] Espinoza-Monroy, M., & De Lafuente, V. (2021). Discrimination of regular and irregular rhythms explained by a time difference accumulation model. *Neuroscience,**459*, 16–26. 10.1016/j.neuroscience.2021.01.035 33549694 10.1016/j.neuroscience.2021.01.035

[CR18] Freestone, D., & Church, R. M. (2016). Optimal timing. *Current Opinion in Behavioral Sciences,**8*, 276–281. 10.1016/j.cobeha.2016.02.031

[CR19] Gardner, J. L. (2019). Optimality and heuristics in perceptual neuroscience. *Nature Neuroscience,**22*(4), 514–523. 10.1038/s41593-019-0340-4 30804531 10.1038/s41593-019-0340-4

[CR20] Gershman, S. J. (2016). Empirical priors for reinforcement learning models. *Journal Of Mathematical Psychology,**71*, 1–6. 10.1016/j.jmp.2016.01.006

[CR21] Gibbon, J. (1977). Scalar expectancy theory and Weber’s law in animal timing. *Psychological Review,**84*(3), 279–325. 10.1037/0033-295X.84.3.279

[CR22] Gibbon, J., Church, R. M., & Meck, W. (1984). Scalar timing in memory. *Annals of the New York Academy of Sciences,**423*(1), 52–77. 10.1111/j.1749-6632.1984.tb23417.x 6588812 10.1111/j.1749-6632.1984.tb23417.x

[CR23] Hachen, I., Reinartz, S., Brasselet, R., Stroligo, A., & Diamond, M. E. (2021). Dynamics of history-dependent perceptual judgment. *Nature Communications,**12*(1), Article 6036. 10.1038/s41467-021-26104-2 34654804 10.1038/s41467-021-26104-2 PMC8521591

[CR24] Hass, J., & Durstewitz, D. (2014). Neurocomputational Models of Time Perception. In H. Merchant & V. de Lafuente (Eds.), *Neurobiology of Interval Timing* (pp. 49–71). Springer. 10.1007/978-1-4939-1782-2_4 10.1007/978-1-4939-1782-2_4 25358705

[CR25] Hass, J., & Durstewitz, D. (2016). Time at the center, or time at the side? Assessing current models of time perception. *Current Opinion in Behavioral Sciences,**8*, 238–244. 10.1016/j.cobeha.2016.02.030

[CR26] JASP Team. (2024). *JASP (Version 0.19.1)[Computer software]* (Version 0.19.1) [Computer software]. https://jasp-stats.org/

[CR27] Jazayeri, M., & Shadlen, M. N. (2010). Temporal context calibrates interval timing. *Nature Neuroscience,**13*(8), 1020–1026. 10.1038/nn.2590 20581842 10.1038/nn.2590 PMC2916084

[CR28] Kiani, R., Hanks, T. D., & Shadlen, M. N. (2008). Bounded integration in parietal cortex underlies decisions even when viewing duration is dictated by the environment. *The Journal Of Neuroscience,**28*(12), 3017–3029. 10.1523/JNEUROSCI.4761-07.2008 18354005 10.1523/JNEUROSCI.4761-07.2008 PMC6670720

[CR29] Killeen, P. R., & Fetterman, J. G. (1988). A behavioral theory of timing. *Psychological Review,**95*(2), 274–295. 3375401 10.1037/0033-295x.95.2.274

[CR30] Klapproth, F. (2018). Towards a Process Model of Temporal Generalization. In A. Vatakis, F. Balcı, M. Di Luca, & Á. Correa (Eds.), *Timing and Time Perception: Procedures, Measures, & Applications*. BRILL. 10.1163/9789004280205

[CR31] Klapproth, F., & Müller, M. (2008). Temporal generalization under time pressure in humans. *The Quarterly Journal of Experimental Psychology,**61*(4), 588–600. 10.1080/17470210701255572 18938277 10.1080/17470210701255572

[CR32] Klapproth, F., & Wearden, J. (2011). Why do temporal generalization gradients change when people make decisions as quickly as possible? *Quarterly Journal of Experimental Psychology,**64*(8), 1646–1664. 10.1080/17470218.2011.564290 21563017

[CR33] Kononowicz, T. W., & Van Rijn, H. (2011). Slow Potentials in Time Estimation: The Role of Temporal Accumulation and Habituation. *Frontiers in Integrative Neuroscience*, *5*. 10.3389/fnint.2011.00048 10.3389/fnint.2011.00048 PMC3171873 21949505

[CR34] Kononowicz, T. W., & Van Rijn, H. (2014). Decoupling interval timing and climbing neural activity: A dissociation between CNV and N1P2 amplitudes. *The Journal of Neuroscience,**34*(8), 2931–2939. 10.1523/JNEUROSCI.2523-13.2014 24553934 10.1523/JNEUROSCI.2523-13.2014 PMC6608524

[CR35] Kononowicz, T. W., Van Wassenhove, V., & Doyère, V. (2022). Rodents monitor their error in self-generated duration on a single trial basis. *Proceedings of the National Academy of Sciences of the United States of America,**119*(9), Article e2108850119. 10.1073/pnas.2108850119 35193973 10.1073/pnas.2108850119 PMC8892352

[CR36] Lebovich, L., Darshan, R., Lavi, Y., Hansel, D., & Loewenstein, Y. (2019). Idiosyncratic choice bias naturally emerges from intrinsic stochasticity in neuronal dynamics. *Nature Human Behaviour,**3*(11), 1190–1202. 10.1038/s41562-019-0682-7 31477911 10.1038/s41562-019-0682-7

[CR37] Maaß, S. C., de Jong, J., van Maanen, L., & van Rijn, H. (2021). Conceptually plausible Bayesian inference in interval timing. *Royal Society Open Science,**8*(8), Article 201844. 10.1098/rsos.201844 34457319 10.1098/rsos.201844 PMC8371368

[CR38] Macar, F., Vidal, F., & Casini, L. (1999). The supplementary motor area in motor and sensory timing: Evidence from slow brain potential changes. *Experimental Brain Research,**125*(3), 271–280. 10.1007/s002210050683 10229018 10.1007/s002210050683

[CR39] Masís, J., Chapman, T., Rhee, J. Y., Cox, D. D., & Saxe, A. M. (2023). Strategically managing learning during perceptual decision making. *eLife,**12*, Article e64978. 10.7554/eLife.64978 36786427 10.7554/eLife.64978 PMC9928425

[CR40] Mathôt, S., Schreij, D., & Theeuwes, J. (2012). Opensesame: An open-source, graphical experiment builder for the social sciences. *Behavior Research Methods,**44*(2), 314–324. 10.3758/s13428-011-0168-7 22083660 10.3758/s13428-011-0168-7 PMC3356517

[CR41] Matthews, W. J., Stewart, N., & Wearden, J. H. (2011). Stimulus intensity and the perception of duration. *Journal of Experimental Psychology: Human Perception and Performance,**37*(1), 303–313. 10.1037/a0019961 20731508 10.1037/a0019961

[CR42] O’Connell, R. G., & Kelly, S. P. (2021). Neurophysiology of human perceptual decision-making. *Annual Review of Neuroscience,**44*(1), 495–516. 10.1146/annurev-neuro-092019-100200 33945693 10.1146/annurev-neuro-092019-100200

[CR43] Ofir, N., & Landau, A. N. (2022). Neural signatures of evidence accumulation in temporal decisions. *Current Biology,**32*(18), 4093-4100.e6. 10.1016/j.cub.2022.08.006 36007527 10.1016/j.cub.2022.08.006

[CR44] Ofir, N., & Landau, A. N. (2025). Motor preparation tracks decision boundary crossing rather than accumulated evidence in temporal decision-making. *The Journal of Neuroscience*. 10.1523/JNEUROSCI.1675-24.2025 40068870 10.1523/JNEUROSCI.1675-24.2025 PMC12019114

[CR45] Özoğlu, E., & Thomaschke, R. (2023). Post-interval potentials in temporal judgements. *Experimental Brain Research,**241*(3), 917–926. 10.1007/s00221-023-06568-y 36806967 10.1007/s00221-023-06568-y PMC9985573

[CR46] Paton, J. J., & Buonomano, D. V. (2018). The neural basis of timing: Distributed mechanisms for diverse functions. *Neuron,**98*(4), 687–705. 10.1016/j.neuron.2018.03.045 29772201 10.1016/j.neuron.2018.03.045 PMC5962026

[CR47] Piras, F., & Coull, J. T. (2011). Implicit, predictive timing draws upon the same scalar representation of time as explicit timing. *PLoS One,**6*(3), Article e18203. 10.1371/journal.pone.0018203 21464972 10.1371/journal.pone.0018203 PMC3064672

[CR48] Prins, N., & Kingdom, F. A. A. (2018). Applying the model-comparison approach to test specific research hypotheses in psychophysical research using the Palamedes toolbox. *Frontiers in Psychology*, *9*(JUL), 1–14. 10.3389/fpsyg.2018.01250 10.3389/fpsyg.2018.01250 PMC6064978 30083122

[CR49] Ratcliff, R. (1979). Group reaction time distributions and an analysis of distribution statistics. *Psychological Bulletin,**86*(3), 446–461. 10.1037/0033-2909.86.3.446 451109

[CR50] Ratcliff, R., Smith, P. L., Brown, S. D., & McKoon, G. (2016). Diffusion decision model: Current issues and history. *Trends in Cognitive Sciences,**20*(4), 260–281. 10.1016/j.tics.2016.01.007 26952739 10.1016/j.tics.2016.01.007 PMC4928591

[CR51] Raviv, O., Ahissar, M., & Loewenstein, Y. (2012). How recent history affects perception: The normative approach and its heuristic approximation. *PLoS Computational Biology,**8*(10), Article e1002731. 10.1371/journal.pcbi.1002731 23133343 10.1371/journal.pcbi.1002731 PMC3486920

[CR52] Schütt, H. H., Harmeling, S., Macke, J. H., & Wichmann, F. A. (2016). Painfree and accurate Bayesian estimation of psychometric functions for (potentially) overdispersed data. *Vision Research,**122*, 105–123. 10.1016/j.visres.2016.02.002 27013261 10.1016/j.visres.2016.02.002

[CR53] Scott, B. B., Constantinople, C. M., Erlich, J. C., Tank, D. W., & Brody, C. D. (2015). Sources of noise during accumulation of evidence in unrestrained and voluntarily head-restrained rats. *eLife*, *4*(DECEMBER2015), 1–23. 10.7554/eLife.11308 10.7554/eLife.11308 PMC4749559 26673896

[CR54] Simen, P., Balcı, F., DeSouza, L., Cohen, J. D., & Holmes, P. (2011). A model of interval timing by neural integration. *Journal of Neuroscience,**31*(25), 9238–9253. 10.1523/JNEUROSCI.3121-10.2011 21697374 10.1523/JNEUROSCI.3121-10.2011 PMC3142662

[CR55] Simen, P., Rivest, F., Ludvig, E. A., Balcı, F., & Killeen, P. (2013). Timescale invariance in the pacemaker-accumulator family of timing models. *Timing and Time Perception,**1*(2), 159–188. 10.1163/22134468-00002018

[CR56] Taatgen, N., & van Rijn, H. (2011). Traces of times past: Representations of temporal intervals in memory. *Memory & Cognition,**39*(8), 1546–1560. 10.3758/s13421-011-0113-0 21626068 10.3758/s13421-011-0113-0 PMC3205264

[CR57] Treisman, M. (1963). Temporal discrimination and the indifference interval. Implications for a model of the “internal clock.” *Psychological Monographs,**77*(13), 1–31. 10.1037/h0093864 5877542 10.1037/h0093864

[CR58] van Maanen, L., & Miletić, S. (2021). The interpretation of behavior-model correlations in unidentified cognitive models. *Psychonomic Bulletin & Review,**28*(2), 374–383. 10.3758/s13423-020-01783-y 32767046 10.3758/s13423-020-01783-y PMC8062378

[CR59] Wagenmakers, E.-J., & Farrell, S. (2004). AIC model selection using Akaike weights. *Psychonomic Bulletin & Review,**11*(1), 192–196. 10.3758/BF03206482 15117008 10.3758/bf03206482

[CR60] Wearden, J. (1991). Human performance on an analogue of an interval bisection task. *The Quarterly Journal of Experimental Psychology B: Comparative and Physiological Psychology,**43B*(1), 59–81. 2017575

[CR61] Wearden, J. (1992). Temporal generalization in humans. *Journal of Experimental Psychology: Animal Behavior Processes,**18*(2), 134–144. 10.1037//0097-7403.23.4.502 9335137

[CR62] Wearden, J. (2004). Decision processes in models of timing. *Acta Neurobiologiae Experimentalis,**64*(3), 303–317. 10.55782/ane-2004-1515 15283474 10.55782/ane-2004-1515

[CR63] Wearden, J., Edwards, H., Fakhri, M., & Percival, A. (1998). Why “Sounds Are Judged Longer Than Lights”: Application of a Model of the Internal Clock in Humans. *Quarterly Journal of Experimental Psychology,**51B*(2), 97–120. 10.1080/713932672 9621837

[CR64] Wearden, J., & Towse, J. N. (1994). Temporal generalization in humans: Three further studies. *Behavioural Processes,**32*(3), 247–264. 24896505 10.1016/0376-6357(94)90046-9

[CR65] Wichmann, F. A., & Hill, N. J. (2001). The psychometric function: I. Fitting, sampling, and goodness of fit. *Perception & Psychophysics,**63*(8), 1293–1313. 10.3758/BF03194544 11800458 10.3758/bf03194544

[CR66] Wichmann, F. A., & Jäkel, F. (2018). Methods in Psychophysics. In *Stevens’ Handbook of Experimental Psychology and Cognitive Neuroscience* (pp. 1–42). John Wiley & Sons, Ltd. 10.1002/9781119170174.epcn507

[CR67] Wiener, M., & Thompson, J. C. (2015). Repetition enhancement and memory effects for duration. *NeuroImage,**113*, 268–278. 10.1016/j.neuroimage.2015.03.054 25818689 10.1016/j.neuroimage.2015.03.054

[CR68] Wiener, M., Thompson, J. C., & Coslett, H. B. (2014). Continuous carryover of temporal context dissociates response bias from perceptual influence for duration. *PLoS One,**9*(6), Article e100803. 10.1371/journal.pone.0100803 24963624 10.1371/journal.pone.0100803 PMC4071004

[CR69] Wilson, R. C., & Collins, A. G. (2019). Ten simple rules for the computational modeling of behavioral data. *eLife,**8*, Article e49547. 10.7554/eLife.49547 31769410 10.7554/eLife.49547 PMC6879303

