# Supplementary material for: A drift-diffusion model of temporal generalization outperforms existing models and captures modality differences and learning effects
Source: Behav Res Methods. 2025 Nov 5;57(12):334. doi: 10.3758/s13428-025-02819-8 (PMC12589256; doi:10.3758/s13428-025-02819-8)
Supplement: Supplementary file 1 — Supplementary file1 (DOCX 565 KB) [file 13428_2025_2819_MOESM1_ESM.docx]

Appendices

Appendix 1: Psychophysical function derivation for the MCG model

The MCG model assumes the following decision rule:

$$-b<\frac{t-s}{t}<b$$

*t* is the true current presented interval. *s* is a memory sample of the standard, drawn from a Gaussian distribution with mean equal to the true standard and standard deviation that grows linearly with standard duration. *b* is a sample of the decision boundary, which is drawn from a Gaussian distribution with a given mean and standard deviation.

Since *t* is positive, and defining ${w_{i}=\left( -1 \right)}^{i}Bt$, where B is the mean boundary, the decision rule can be rewritten as:

$$w_{1}<t-s<w_{2}$$

The probability of a “same” response is then

$$P\left( "same" \right)=P\left( t-s<w_{2} \right)-P\left( t-s<w_{1} \right)$$

Equivalent to

$$P\left( "same" \right)=P\left( t-s-w_{2}<0 \right)-P\left( t-s-w_{1}<0 \right)$$

The left-hand side within each parenthesis is a sum of independent Gaussian random variables (*s* and *w­_i_*) and a constant (*t*). Hence, that sum is itself a Gaussian random variable with the following mean and variance (*S* and *B* are the mean of memory and boundary distributions, respectively):

$$E\left( t-s-w_{i} \right)=t-S-\left( -1 \right)^{i}bt=t\left( 1-\left( -1 \right)^{i}Bt \right)-S$$

$$Var\left( t-s-w_{i} \right)=Var\left( w_{i} \right)+Var\left( s \right)=t^{2}{\sigma_{B}}^{2}+S^{2} \gamma^{2}$$

With this, we can write the psychophysical function:

$$P\left( \text{same}|t;k,B,\sigma_{B} \right)=\Phi\left( z_{2} \right)-\Phi\left( z_{1} \right)$$

$$z_{i}=\frac{t\left( 1+\left( -1 \right)^{i}B \right)-S}{\sqrt{t^{2}{\sigma_{B}}^{2}+S^{2} k^{2}}}$$

Appendix 2: Psychophysical function derivation for the double-boundary DDM

The mathematical formulation of our model is as follows (adapted from Simen et al., 2011): A drift-diffusion process, representing the momentary perceived duration (accumulated temporal evidence), starts at the stimulus onset. The dynamics of the DV is given by:

$$dx=A\cdot dt+w\cdot dB$$

Where *x* is the DV, *A* is the drift rate (how fast the DV grows over time on average), $w\cdot dB$ represents the variability in momentary DV (the noise level). The probability density function of DV values at a given moment *t* is given by the following Gaussian distribution:

$$p\left( x|t \right)=\frac{1}{w\sqrt{t2\pi}}exp\left( -\frac{1}{2}\left( \frac{x-At}{w\sqrt{t}} \right)^{2} \right)$$

We define two additional parameters, *z_l_* and *z_u_*, which represent the lower and upper decision boundaries, respectively. The probability of a “same” response for an in interval of duration *t* is the probability that the DV at moment *t* is between the boundaries:

$$p\left( "same"|t;A,w,z_{l},z_{u} \right)=\frac{1}{w\sqrt{t2\pi}}\int_{z_{l}}^{z_{u}} exp\left( -\frac{1}{2}\left( \frac{x-At}{w\sqrt{t}} \right)^{2} \right)dx$$

We perform the following change of variables, and define $\Phi\left( x \right)$ as the cumulative standardized Gaussian function evaluated at $x$:

$$x'=\frac{x-At}{w\sqrt{t}}, dx'=\frac{1}{w\sqrt{t}}dx$$

$$x'\left( z_{l} \right)=\frac{z_{l}-At}{w\sqrt{t}}, x'\left( z_{u} \right)=\frac{z_{u}-At}{w\sqrt{t}}$$

$$p\left( "same"|t;A,w,z_{l},z_{u} \right)=\frac{1}{\sqrt{2\pi}}\int_{\frac{z_{l}-At}{w\sqrt{t}}}^{\frac{z_{u}-At}{w\sqrt{t}}} exp\left( {-\frac{x'}{2}}^{2} \right)dx'$$

$$p\left( "same"|t;A,w,z_{l},z_{u} \right)=\Phi\left( \frac{z_{u}-At}{w\sqrt{t}} \right)-\Phi\left( \frac{z_{l}-At}{w\sqrt{t}} \right)$$

In our data it is not possible to estimate all four parameters since the boundaries and drift coefficient can always offset the effect of changing the drift rate. Therefore, we define three new parameters which are ratios of the diffusion and boundaries against the drift rate: $b_{l}=\frac{z_{l}}{A}, b_{u}=\frac{z_{u}}{A}, c=\frac{w}{A}$), which brings us to the final psychophysical function:

$$p\left( "same"|t;b_{l},b_{u},c \right)=\Phi\left( \frac{b_{u}-t}{c\sqrt{t}} \right)-\Phi\left( \frac{b_{l}-t}{c\sqrt{t}} \right)$$

Supplemental Data


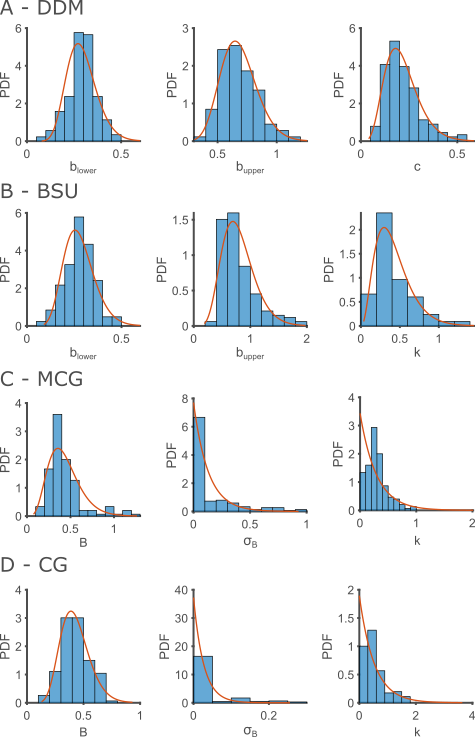


Figure S1: Parameter distribution estimated from both experiments. Histograms show the estimated values, and red curves depict the parametric distribution fitted to each histogram.


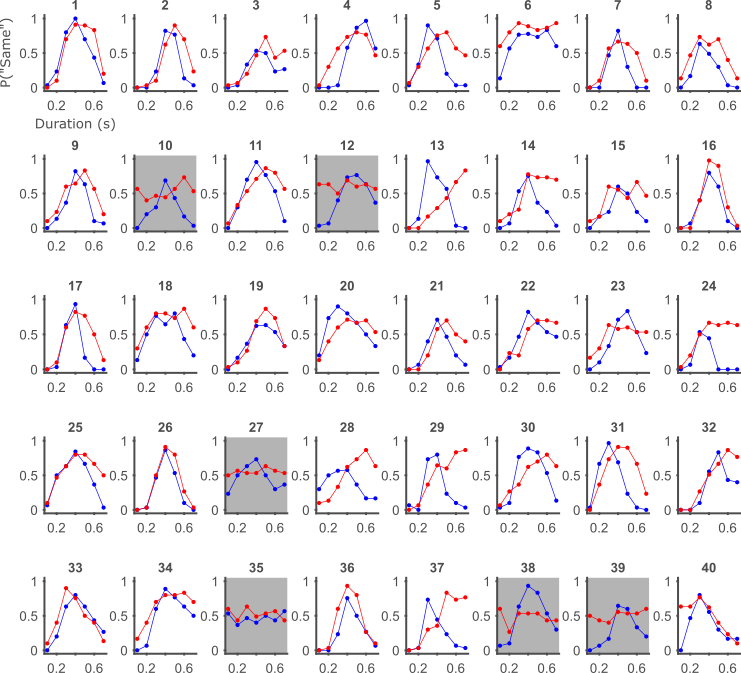


Figure S2: Single participants data, auditory-visual experiment. Color marks the modality (blue for auditory and red for visual). Participants with gray background were excluded from the analysis.


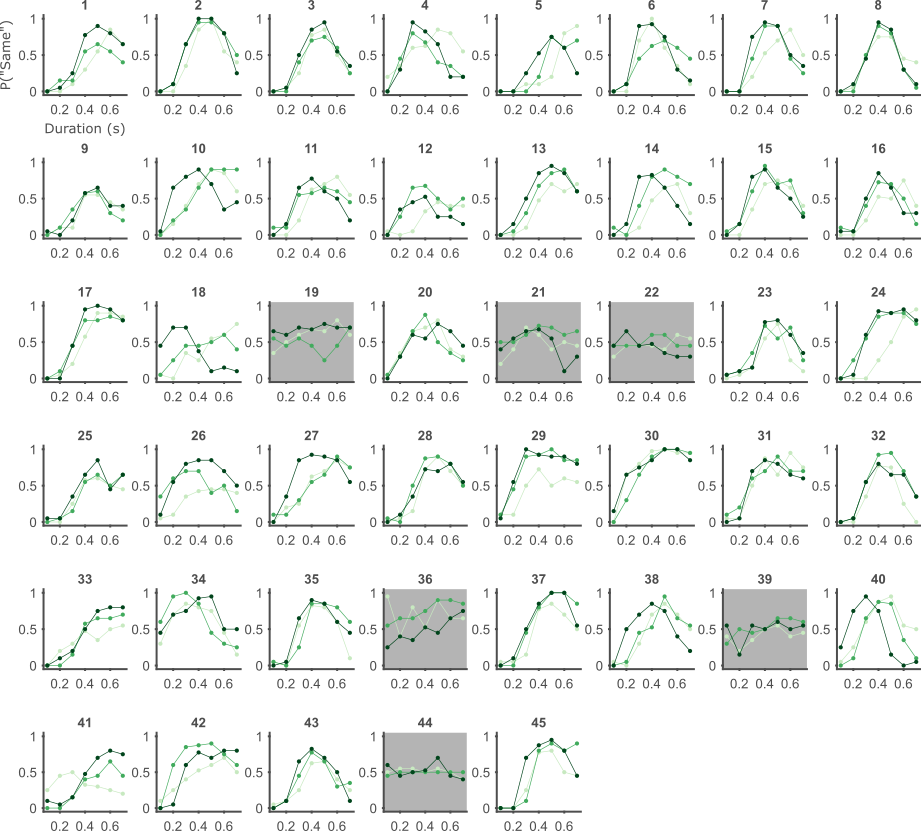


Figure S3: Single participants data, learning experiment. Color hue marks the tertile (light for early in the experiment and dark for late). Participants with gray background were excluded from the analysis.
